# Editing inducer elements increases A-to-I editing efficiency in the mammalian transcriptome

**DOI:** 10.1186/s13059-017-1324-x

**Published:** 2017-10-23

**Authors:** Chammiran Daniel, Albin Widmark, Ditte Rigardt, Marie Öhman

**Affiliations:** Department of Molecular Biosciences, The Wenner-Gren Institute, Stockholm University, Svante Arrheniusväg 20C, 10691 Stockholm, Sweden

**Keywords:** RNA editing, Adenosine deamination, Glutamate receptor, ADAR, EIE, miRNA

## Abstract

**Background:**

Adenosine to inosine (A-to-I) RNA editing has been shown to be an essential event that plays a significant role in neuronal function, as well as innate immunity, in mammals. It requires a structure that is largely double-stranded for catalysis but little is known about what determines editing efficiency and specificity in vivo. We have previously shown that some editing sites require adjacent long stem loop structures acting as editing inducer elements (EIEs) for efficient editing.

**Results:**

The glutamate receptor subunit A2 is edited at the Q/R site in almost 100% of all transcripts. We show that efficient editing at the Q/R site requires an EIE in the downstream intron, separated by an internal loop. Also, other efficiently edited sites are flanked by conserved, highly structured EIEs and we propose that this is a general requisite for efficient editing, while sites with low levels of editing lack EIEs. This phenomenon is not limited to mRNA, as non-coding primary miRNAs also use EIEs to recruit ADAR to specific sites.

**Conclusions:**

We propose a model where two regions of dsRNA are required for efficient editing: first, an RNA stem that recruits ADAR and increases the local concentration of the enzyme, then a shorter, less stable duplex that is ideal for efficient and specific catalysis. This discovery changes the way we define and determine a substrate for A-to-I editing. This will be important in the discovery of novel editing sites, as well as explaining cases of altered editing in relation to disease.

**Electronic supplementary material:**

The online version of this article (doi:10.1186/s13059-017-1324-x) contains supplementary material, which is available to authorized users.

## Background

Complex organisms require a great diversity of gene products for proper development and function, particularly in the brain. This is achieved by the use of numerous co- or post-transcriptional processes, such as alternative splicing, alternative polyadenylation, and RNA editing. Adenosine-to-inosine (A-to-I) RNA editing is a highly conserved RNA modification process that occurs in all metazoan lineages [1]. Inosine base pairs with C and is interpreted as G by the cellular machineries. Hence, A-to-I RNA editing can be designated as an A-to-G conversion and, if situated in exonic sequence, it has the potential to alter codons and consequently contribute to the expression of additional protein isoforms (reviewed in [2]). A-to-I conversions within introns and 3′ UTRs can also have an impact on the transcriptome, e.g., by creating new splice sites and changing miRNA target recognition. A-to-I editing is essential to the organism and aberrant editing has been linked to a variety of different human diseases: amyotrophic lateral sclerosis (ALS) and other neurological disorders, several types of cancer, and autoimmune disorders such as the Aicardi-Goutières syndrome (AGS) [3–6]. To understand what determines the level of editing in different substrates and under different circumstances, we need to know the mechanism of substrate recognition. It is, however, still largely unclear what factors determine the efficiency of editing.

A-to-I RNA editing is performed by the adenosine deaminases that act on RNA (ADAR) enzymes that recognize adenosines located in double-stranded RNA (dsRNA) to be deaminated into inosines [7]. ADAR proteins are evolutionarily conserved in metazoans and mammals have two enzymatically active ADAR enzymes, ADAR1 and ADAR2 [8–10]. In some cases, the substrate selectivity of the two enzymes overlaps, but more commonly the targets are specific for either enzyme [11–13]. ADAR1 and ADAR2 share certain domain structures, such as the deaminase domain and the double-stranded RNA binding domains (dsRBDs). However, the numbers of dsRBDs differ between the two enzymes (ADAR1 contains three while ADAR2 contains two) as well as the spacing between them. The dsRBDs recognize one face of the sugar backbone of an A-form helix, such as the RNA duplex, spanning two minor grooves and an intervening major groove [14]. Thus, there is little sequence specificity via interaction of the dsRBDs and theoretically they can interact with any double-stranded RNA longer than 16 nucleotides (nt). However, sequence-specific interactions between the two dsRBDs of human ADAR2 at the GluA2 stem loop at the R/G site have been reported based on the NMR structure [15]. Interestingly, it has recently been shown that the deaminase domain also requires a double-stranded structure in order to interact with the substrate and perform the catalysis [16, 17].

In general, there are two categories of A-to-I RNA editing determined by the structure of the RNA. Long double-stranded structures, commonly found in introns and 3′ UTRs, are subjected to hyper-editing of many adenosines in what appears to be a random manner [18–21]. Most of this type of editing occurs within inverted repeat elements, commonly within introns and non-coding sequences. This is also the most common A-to-I editing event and human next-generation sequencing together with advanced computational methods has predicted up to 100 million sites [22, 23]. The other, more site-selective category is often present in shorter duplexes interrupted by bulges and internal loops and commonly occurs within coding regions. These duplexes are often formed by base pairing between the exon sequence containing the editing site and an adjacent intron. Site-selective A-to-I RNA editing is highly conserved and particularly prone to cause amino acid changes with functionality in neurotransmission-related genes (reviewed in [2]). One of the most prominent selectively edited sites is located in the brain-specific GluA2 transcript, coding for the AMPA glutamate receptor. The Q/R site in GluA2 is ADAR2-specific and edited in almost 100% of all transcripts in the adult mammalian brain [24]. Editing at this site results in an amino acid change from glutamine (Q) to arginine (R) that reduces the receptor permeability to Ca^2+^ [25]. This editing event is crucial for normal brain development and function, as shown by studies on ADAR2-deficient mice. These mice develop severe epileptic seizures and die within three weeks after birth, mainly due to the lack of editing at the Q/R site [26]. However, it is still not fully elucidated why the Q/R site is so remarkably prone to editing.

Another highly edited site changes a codon for isoleucine to methionine (I/M) in the Gabra-3 transcript, coding for the α3 subunit of the GABA_A_ receptor [27]. We have previously shown that editing at the I/M site in exon 9 of Gabra-3 requires a conserved 149-nt long intronic sequence located downstream of the I/M editing site for efficient editing [28]. This intronic editing inducer element (EIE) forms a long double-stranded structure prone to hyper-editing.

In the present study, we show that the efficiency of editing at the GluA2 Q/R site is dependent on a conserved 45 base pair (bp) long intronic stem structure acting as an EIE. The EIE of GluA2 can also induce editing at other selectively edited sites and even increase the efficiency of editing at sites of low efficiency. We further show that EIEs are commonly present adjacent to efficiently edited sites, while they are absent in proximity of sites of low editing efficiency. Moreover, EIEs are not limited to sites within coding sequence; editing within non-coding RNA such as pri-miRNAs can also be induced by EIEs. We therefore suggest that the use of EIEs is a general mechanism used by the enzyme to increase both editing specificity and efficiency.

## Results

### Editing at the Q/R site in GluA2 requires an editing inducer element

**Fig. 1:**
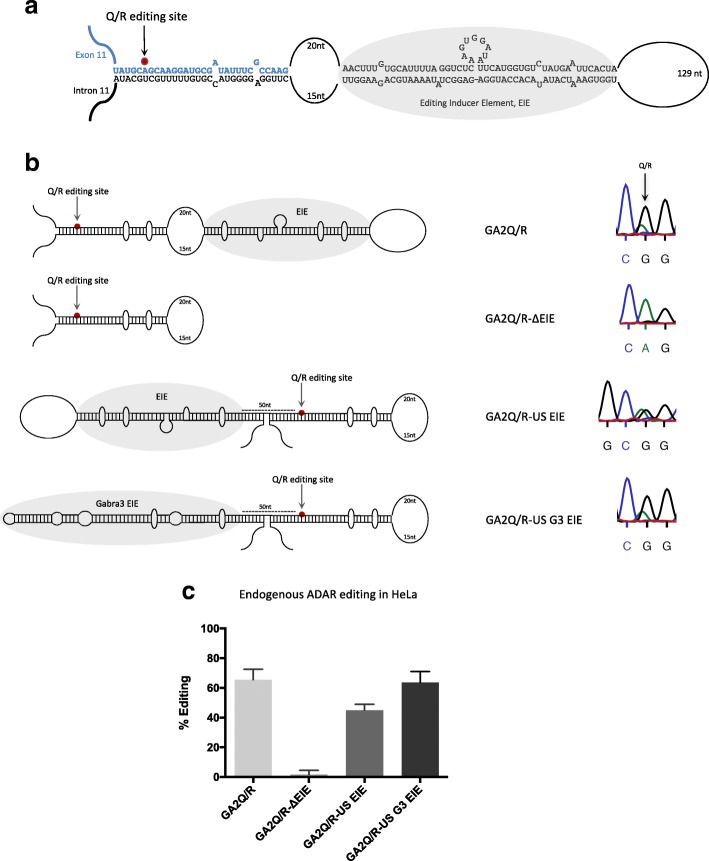
Structural requirements for efficient editing at the Q/R site of the GluA2 transcript. **a** GluA2 RNA structure at exon11–intron11. Exonic sequence is illustrated in *blue* and intronic sequence in *black*. The Q/R site is located in exon 11 and indicated with a *red dot*. The region in *grey* illustrates the position of the EIE, 45 nt downstream of the Q/R site. **b** *Left*: the wild-type construct, GA2Q/R containing the Q/R editing site and the EIE; the GA2Q/R-ΔEIE mutant where the EIE has been deleted; the GA2Q/R-US EIE where the EIE has been moved to a position 50 nt upstream of the Q/R site; and GA2Q/R-US G3 EIE where the Gabra-3 EIE is placed 50 nt upstream of the Q/R site. *Right*: sequencing chromatograms illustrating editing of the different GluA2 reporters by endogenous ADAR2 in HeLa cells. **c** Quantification of editing efficiency at the Q/R site from the different GA2Q/R constructs in HeLa cells. The mean value of the ratio between the A and G peak heights from three individual experiments is calculated as the percentage of editing. *Error bars* are standard deviation

A-to-I editing is exceptionally efficient at the Q/R site of the transcript coding for glutamate receptor subunit GluA2 [29]. The Q/R site is situated in a stem loop structure consisting of two duplexes separated by a larger internal loop of 35 nucleotides (nt; Fig. 1a). The edited Q/R site is located in the shorter stem consisting of exon 11 and an editing complementary sequence (ECS) that is part of the downstream intron. This duplex contains a predicted 28 bp with two mismatched bulges. Downstream of this stem, separated by a larger internal loop, there is a longer stem consisting of 43 bp, interrupted by four mismatches and a bulge of 8 nt. It has recently been shown that a duplex of about 20 bp is sufficient for the deaminase domain of ADAR2 to interact and perform the catalysis [16, 17]. Thus, the 28-bp duplex holding the Q/R site should be sufficient for the deamination catalysis. To determine the requirements for efficient editing at the Q/R site we investigated the contribution of the downstream stem. Editing reporter constructs were created expressing transcripts containing the wild-type sequence with both stem structures (GAQ/R) and only the shorter stem with the Q/R site (GAQ/R-ΔEIE) (Fig. 1b). These reporters were transfected into HeLa cells, utilizing the endogenous ADAR2 enzyme, as well as co-transfected with transient ADAR2 in HEK293 cells (Additional file 1: Figure S1). Editing was determined by measuring the peak heights (A and G) in the chromatogram after Sanger sequencing of extracted total RNA after RT-PCR. On average, 66% of the GAQ/R transcripts were edited by the endogenous editing enzyme, while no editing could be detected in the absence of the longer downstream stem structure (Fig. 1b, c). We have previously shown that several other exonic editing sites depend on editing inducer elements (EIE) for efficient editing [28, 30]. These EIEs are stem loop structures located either upstream or downstream of the site-selectively edited duplex. We speculated that the 45-bp long stem, deleted in the GAQ/R-ΔEIE construct, either helps to stabilize the shorter stem by extending the stem loop structure or functions as an EIE for efficient editing of the Q/R site in the GluA2 transcript. If the 45-bp long stem in the GluA2 transcript functions as an EIE rather than stabilizing the structure, the location of this stem, upstream or downstream, should be independent of its capability to induce editing. Indeed, placing the 45-bp stem 50 nucleotides upstream of the Q/R site (GAQ/R-US EIE) rescued Q/R editing in HeLa cells (Fig. 1b, c). Furthermore, we have previously shown that the EIE in the Gabra-3 transcript can induce editing independently of its location upstream or downstream of the I/M editing site. We therefore replaced the EIE of GluA2 with the EIE from the Gabra-3 transcript. Indeed, the Gabra-3 EIE, placed upstream of the Q/R site, could induce editing (GAQ/-US G3 EIE) to the same level as the wild-type sequence (Fig. 1b, c). During transient co-transfection of an ADAR2 in HEK293 cells, approximately 80% of the transcripts were edited in GA2Q/R, while GAQ/R-ΔEIE had less than 50% editing (Additional file 1: Figure S1). As previously described by us and others, no endogenous editing is detected in HEK293 cells in any transiently expressed editing reporter substrate (data not shown). Upstream EIEs could rescue the editing levels and resulted in highly efficient editing also with transient ADAR2. These results indicate that the downstream 45-bp stem functions as an EIE rather than stabilizing the stem in the immediate vicinity of the Q/R editing site and the editing induction is independent of the location of the EIE and its specific sequence.

The Q/R site of GluA2 has been shown to be highly edited in the brain during early embryogenesis, while most other sites show low editing levels at this stage. We speculated that the high level of editing at the Q/R site of GluA2 in the embryo might be explained by this being a high affinity site for ADAR2, requiring a lower amount of the editing enzyme for full catalysis compared to other sites. We wanted to investigate if the EIE contributes to the highly efficient editing at the Q/R site by attracting ADAR2 to the transcript. If so, a lower concentration of the ADAR2 enzyme should be required for efficient editing in the presence of the EIE compared to its absence. A titration of the ADAR2 expression vector (0–1.25 μg) was transfected into HEK293 cells together with a constant concentration (0.75 μg) of the GAQ/R or GAQ/R-ΔEIE reporter. Indeed, only 0.1 μg of transfected ADAR2 expression vector was enough to reach 83% editing in a co-transfected GA2Q/R reporter, while only 49% editing, on average, was seen in the GAQ/R-ΔEIE reporter using the same amount of transfected ADAR vector (Additional file 1: Figure S2). Eventually, at transfection of 1.25 μg ADAR2 expression vector, editing of the GAQ/R-ΔEIE transcripts reached similar levels (79%) as that of GAQ/R transcripts (87%) (Additional file 1: Figure S2). The ADAR2 enzyme is then assumed to be present in large excess. In summary, these results indicate that the EIE contributes to the high affinity editing at the Q/R site of GluA2, possibly by attracting the editing enzyme and thereby increasing the local concentration of ADAR2 to promote editing at the Q/R site.

### The EIE of GluA2 can induce editing by both ADAR1 and ADAR2

**Fig. 2:**
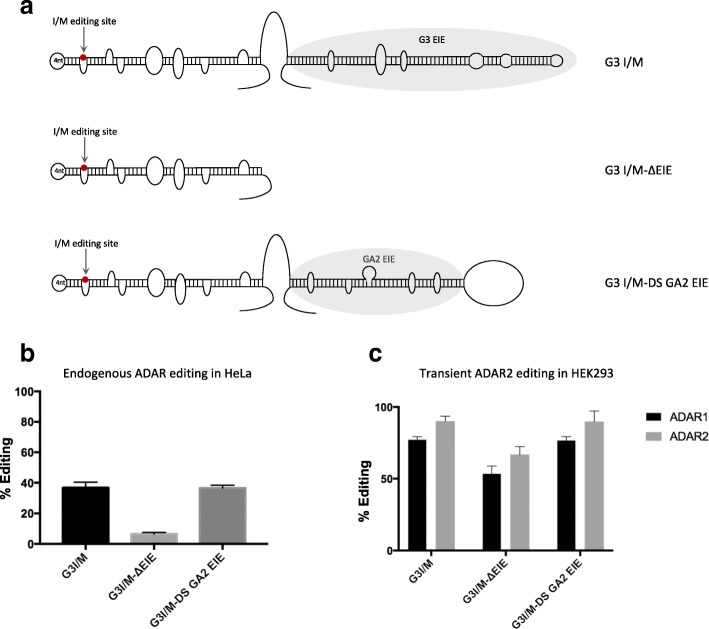
The EIE of GluA2 can induce editing at the IM site in Gabra-3. **a** The wild-type Gabra-3 construct (G3 I/M) showing the short stem structure formed at the edited I/M site (*red dot*) and the EIE (*in grey*); the G3 I/M-ΔEIE mutant were the EIE has been deleted; and G3 I/M-DS GA2 EIE were the Gabra-3 EIE is replaced by the GluA2 EIE. **b** Quantification of editing efficiency at the I/M site in the different G3I/M constructs transfected into HeLa cells. **c** Quantification of editing efficiency at I/M site from the different G3I/M constructs when co-transfected with ADAR1 or ADAR2 in HEK293 cells. The mean value of the ratio between the A and G peak heights from three separate experiments was calculated as percentage editing. *Error bars* are standard deviation

If the 45-bp stem downstream of the Q/R site in GluA2 is an EIE, it should be able to induce editing also at other ADAR editing sites. In mouse brain, over 90% of the Gabra-3 transcripts are edited at the I/M site [31]. We showed previously that editing at the I/M site of Gabra-3 is dramatically reduced in the absence of its EIE [28]. We replaced the confirmed EIE of Gabra-3, located downstream of the I/M editing site, with the EIE from GluA2 (Fig. 2a). Here, we confirm previous results demonstrating that editing at the I/M site of a Gabra-3 reporter (G3 I/M) is reduced from 40% to less than 10% in the absence of the EIE in HeLa cells expressing endogenous ADARs (G3 I/M ΔEIE) (Fig. 2b). Placing the GluA2 EIE downstream of the stem with the I/M site (G3 I/M DS GA2 EIE) rescued editing and gave a similar I/M editing level as the wild-type Gabra-3 reporter (Fig. 2b). This result indicates that the EIE of GluA2 works efficiently as an inducer of editing also in other substrates.

The I/M site of Gabra-3 has previously been shown to be a substrate for editing by both ADAR1 and ADAR2 [27], while the Q/R site of GluA2 is exclusively edited by ADAR2 [11]. To determine if the GluA2 EIE could work as a recruitment element for both ADAR1 and ADAR2, the Gabra-3 I/M editing reporter construct with the downstream GluA2 EIE was co-expressed with either ADAR1 or ADAR2 in HEK293 cells and compared to the editing efficiency in the other reporters (Fig. 2c). As previously shown, Gabra-3 is edited by both ADAR1 and ADAR2 and the GluA2 EIE is able to induce editing of the I/M site by both enzymes to similar levels as the wild type EIE from Gabra-3 (Fig. 2c). These results suggest that the GluA2 EIE can work as an efficient recruitment element for both ADAR1 and ADAR2.

### An internal loop separating the EIE from the Q/R site in GluA2 sets selectivity

**Fig. 3:**
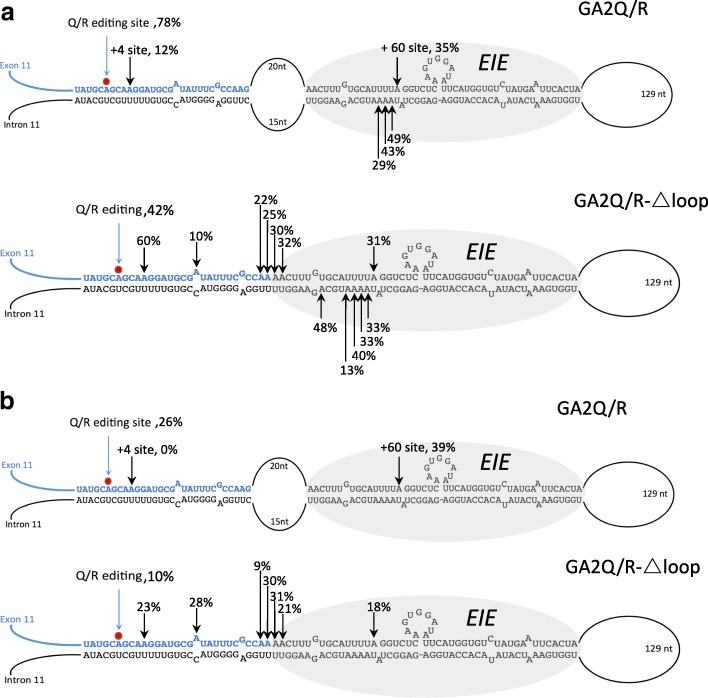
Selectivity of editing at the Q/R site in GluA2. **a** *Top*: sites of editing and average percentage editing in the GluA2 reporter GA2Q/R co-transfected with an ADAR2 expression vector in HEK293 cells. Exon sequence is indicated in *blue* and the editing inducer element (EIE) shaded in *grey*. The Q/R site is indicated with a *red dot. Bottom*: sites of editing in the GluA2 reporter with the internal loop deleted (GA2Q/R-Δloop) co-transfected with ADAR2 in HEK293 cells. **b** *Top*: sites of editing and average percentage editing in the GluA2 reporter GA2Q/R co-transfected with the mutant ADAR2-EAA-E488Q expression vector in HEK293 cells. *Bottom*: sites of editing in the GluA2 reporter with the internal loop deleted (GA2Q/R-Δloop) co-transfected with ADAR2-EAA-E488Q in HEK293 cells. The mean value of the ratio between the A and G peak heights from three separate experiments was calculated as percentage editing

The EIE in GluA2 is separated from the stem containing the selective Q/R site by a large internal loop (Fig. 1a) that may function as a border to separate ADAR recruitment from editing site specificity and efficiency. To investigate if the internal loop plays a role in Q/R site specificity, we removed the loop of 35 nt from the substrate, fusing the Q/R stem with the EIE stem in the editing reporter (GA2Q/R-Δloop) (Fig. 3a). As presented above, transient ADAR2 in HEK293 cells edited the Q/R site in 78% of the wild-type reporter. Five other editing sites, previously shown to be edited in vivo [32], were detected at +4, +60, +261, +262, and +263 from the Q/R site. These were edited in 12, 35, 49, 43, and 29% of the transcripts, respectively (Fig. 3a). Removal of the internal loop, by deleting 37 nt from +24 to +44 and +276 to +291 (GA2Q/R-Δloop), resulted in a dramatic change in both editing efficiency and specificity. The most highly edited site was +4 with 60% editing, while the Q/R site was edited in only 42%, on average, of the transcripts (Fig. 3a). Seven new sites were also detected in the transcript with editing efficiency from 10 to 48%. These edited adenosines are located on both strands in the long, extended stem structure. The change in editing efficiency and specificity after the removal of the internal loop was also seen in HeLa cells expressing endogenous ADAR (data not shown). This result reveals that the internal loop limits the number of edited adenosines in the vicinity of the Q/R site, but it also contributes to editing efficiency at the Q/R site.

We next investigated the role of the double-stranded RNA binding domains (dsRBDs) for editing specificity and selectivity. Mutations from KKxxK to EAxxA in the two dsRBDs of ADAR2 were made to impair their RNA interaction as previously described [33]. To compensate for the inefficient editing achieved with this mutant (data not shown), we increased the catalytic rate by a single mutation in the catalytic domain—E488Q amino acid change—as previously shown [34]. This mutation has been shown not to affect RNA binding in vitro. This ADAR2-EAA-E488Q mutant expression vector was co-transfected with the GluA2 Q/R editing reporter (GA2Q/R) in HEK293 cells. Although editing was much more inefficient at the Q/R site compared to using the wild-type enzyme—from 78 to 26% on average—the site selectivity persisted (Fig. 3b). Interestingly, the efficiency of editing at the +60 site in the intron sequence was unaffected by the mutations (Fig. 3b). These editing levels may indicate that efficient editing can be achieved without the contribution of the dsRBDs. Removing the internal loop in the transcript (GA2Q/R-Δloop) gave rise to promiscuous editing in a similar way as with the wild-type enzyme, although five sites in the intronic EIE were missing. As a control, we also introduced E488Q as a single mutation in ADAR2. ADAR2-E488Q increased editing at several sites in GA2Q/R compared to wild type, and the specificity was lost in a similar way as with ADAR2 wild -type after removal of the internal loop in co-transfections with the GA2Q/R-Δloop (Additional file 1: Figure S3a). Equal expression of the transiently expressed ADAR2 mutants and wild -type was analyzed by western blot (Additional file 1: Figure S3b). In conclusion, this result suggests that a basic low level of editing can be achieved without the contribution of the dsRBDs and that the deamination domain determines the selectivity. Furthermore, the dsRBDs are required to achieve editing of high efficiency at the Q/R site.

### An EIE induces editing at the Q/R site in kainate receptor subunit GluK2

**Fig. 4:**
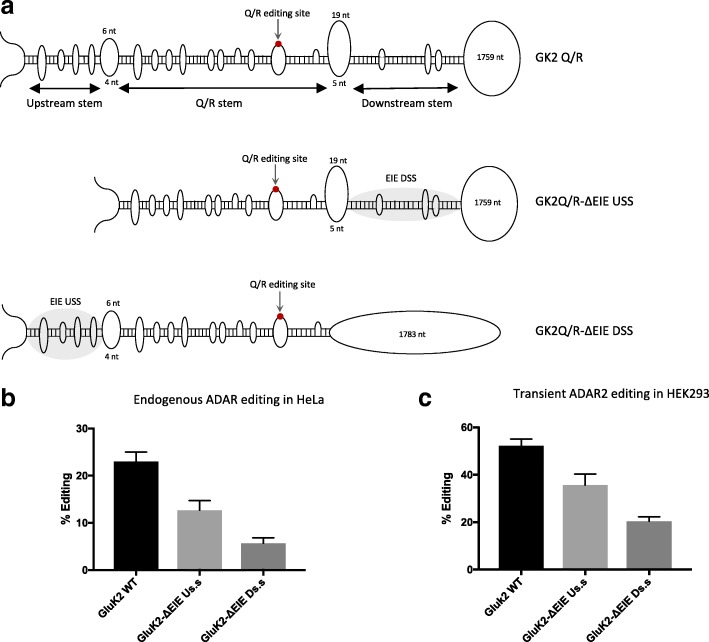
Structural requirements for efficient editing at the Q/R site in the GluK2 transcript. **a** *Top*: the GK2Q/R construct showing the structure formed in the vicinity of the Q/R site. The edited adenosine (*red dot*) is located in exon 12 and pairing sequences are located within intron 12. Three stems—the *Upstream stem*, *Q/R stem*, and *Downstream stem*—are separated by larger internal loops. The region in *grey* illustrates the position of the EIE. *Middle*: the GK2Q/R-ΔEIE USS reporter where the upstream stem has been deleted. *Bottom*: the GK2Q/R-ΔEIE DSS reporter where the downstream stem has been disrupted. **b** Quantification of editing efficiency at the Q/R site from the different GK2Q/R constructs transfected into HeLa cells. **c** Quantification of editing efficiency at the Q/R site from the different GK2Q/R constructs co-transfected with ADAR2 in HEK 293 cells. The mean value of the ratio between the A and G peak heights from three individual experiments was calculated as the percentage of editing. *Error bars* are standard deviation

The finding that a dsRNA stem structure, separated from the specific editing site by a larger internal loop, can work as an editing inducer made us look for EIEs in the vicinity of other highly edited ADAR substrates. In the kainate receptor subunit GluK2, over 90% of the transcripts are edited at the Q/R site in several different brain regions [35]. As in GluA2, the RNA secondary structure in the vicinity of the Q/R site in the GluK2 transcript is formed by exon and intron sequences, although both structure and sequence differ between the two transcripts. GluK2 has three stem regions separated by internal loops in the vicinity of the Q/R site (Fig. 4a). The stem holding the Q/R site in GluK2 is formed with an ECS located in the intron, 1885 nt downstream. Two flanking stem structures are separated from the edited stem (Q/R stem) by two larger internal loops. To investigate if the stems flanking the Q/R stem are required for efficient editing at the Q/R site in GluK2, editing reporters were made that contain the wild-type GluK2 exon and downstream intron sequence (GK2Q/R), a deletion of the downstream stem (GKQ/R-ΔEIE DSS), and a deletion of the upstream stem (GK2Q/R-ΔEIE USS) (Fig. 4a). In HeLa cells, 23% of the transgenic wild-type transcripts (GK2Q/R) were edited by endogenous ADAR. Deleting the stem upstream of the Q/R site (GluK2-ΔEIE USS) led to a decrease in editing by 50%, while disruption of the downstream stem (GluK2-ΔEIE DSS) had an even more severe effect on editing, with only 5% of the transcripts being edited (Fig. 4b). Transient ADAR2 co-transfected in HEK293 cells showed the same trend of editing efficiency. The wild-type GluK2 sequence was edited in an average of 52% of the transcripts, while editing decreased to 35% in GluK2-ΔEIE USS and down to 20% in GluK2-ΔEIE DSS (Fig. 4c). These results demonstrate that efficient editing at the Q/R site in GluK2, just like the Q/R site in GluA2, requires adjacent stem structures functioning as editing inducer elements. Moreover, even though both of these stem structures contribute to the increasing efficiency of editing at the Q/R site in GluK2, the downstream stem appears to play the major role as an EIE.

### Conserved EIEs are found close to efficiently edited sites

We hypothesized that the sites with low editing efficiency lack adjacent dsRNA structures that could function as EIEs, while the sites showing high editing efficiency are flanked by conserved double-stranded structures, functioning as ADAR recruitment elements. To investigate this hypothesis, we analyzed substrates with conserved site selective editing and looked for the presence of stable, conserved stem structures in the vicinity. In total, 23 substrates were analyzed with sites of editing ranging from 7–100% (Table 1). The substrates were chosen based on conserved, experimentally verified site-selective editing within coding sequence which results in amino acid changes after translation (for references see Table 1). Site selective editing was categorized into two groups: i) highly efficient editing of 50–100% in adult tissue; and ii) sites of consistent low editing efficiency of 1–45%. All but one of our chosen sites were located within coding sequence, creating an amino acid change upon editing. The only exception was the pre-mRNA of ADAR2 with several intronic sites, where one of them (+1) creates an alternative 3′ splice site [36]. The structures in the vicinity of the edited site were analyzed using mfold [37] to look for the presence of conserved sequences adjacent to the edited site, but not directly part of the sequence at the editing site or its ECS, that could possibly form stable stem structures. In concert with the mfold results, RNAfold from the ViennaRNA Package 2.0 [38] was used to predict stable dsRNA structures formed by the conserved sequences. Strikingly, 10 out of 11 sites with an editing efficiency of 50% and above had conserved sequences with the ability to form stable dsRNA structures adjacent to the edited stem (Table 1; Additional file 2: Figure S5). The majority of these stems consists of unusually conserved intronic sequences. To investigate if the identified conserved EIEs keep the duplex structure by compensatory mutations, sequence alignments were done between mouse and human. Indeed, as shown in Additional file 1: Figure S4, in the EIE of GluA2 and GluK2 both structure and sequence are highly conserved and the few nucleotides that differ between the species are located in bulges or preserve the double strand. Furthermore, edited adenosines are detected in these regions, indicating the presence of the ADAR enzyme. In addition we have previously shown that the EIE of Gabra-3 also is conserved and edited [28].

**Table 1:** Conserved site selective editing in mammals

Substrate	Edit site^a^	Percentage editing	Reference to editing level	Conserved adjacent stem^b^	Stable adjacent stem^c^	Length of EIE (nt)	Number of base pairs in EIE	Distance edit site–EIE (nt)	5′ EIE	3′ EIE
GluA2	Q/R	100	[31]	Yes	Yes	102	43	45		X
Gabra3	I/M	92	[31]	Yes	Yes	149	54	143		X
GluA3	R/G	91	[31]	Yes	Yes	81	32	220		X
FLNB	Q/R	90	[52]	No	ND	ND	ND	ND		
Htrc2	I/V	85	[31]	Yes	Yes	64	27	159		X
GluK2	Q/R	83	[31]	Yes	Yes	129, 60	42, 25	48, 32	X	X
ADAR2	+24	82	[31]	Yes	Yes	86	37	60	X	
Cyfip2	K/E	75	[31]	Yes	Yes	116	47	138		X
GluA2	R/G	72	[31]	Yes	Yes	79	30	230		X
GluK1	Q/R	62	[31]	Yes	Yes	73	27	70		X
BLCAP	Y/C	50	[53]	Yes	Yes	71, 59, 64	33, 26, 28	37, 90, 123	X	
IGFBP7	K/E	45	[54]	No	ND	ND	ND	ND		
FLNA	Q/R	43	[31]	Yes	Yes	105	36	38		X
Nova1	S/G	30	[47]	No	ND		ND	ND		
KCNA1	I/V	25	[31]	No	ND		ND	ND		
PLCH2	R/G	20	[47]	No	ND		ND	ND		
TMEM63B	Q/R	20	[47]	No	ND		ND	ND		
CCNI	R/G	15	[47]	No	ND		ND	ND		
Azin1	S/G	10	[52]	No	ND		ND	ND		
Copa	I/V	10	[52]	No	ND		ND	ND		
GPATCH8	K/R	10	[47]	No	ND		ND	ND		
NCSTN	S/G	7	[47]	No	ND		ND	ND		
OSGEP	I/M	7	[47]	No	ND		ND	ND		

^a^Amino acid change after A-to-I editing

^b^Conserved stem adjacent to edited site, predicted from mfold [37]

^c^Stable adjacent stem, as predicted from RNAfold, where high base-pairing probabilities are calculated from the minimum free energy of single sequences [38]

*ND* not determined

Among the 12 sites with an editing efficiency of 45% and below, only the Q/R site of FLNA was flanked by a conserved sequence with the potential to form a stable adjacent stem. This site has been shown to be edited in an average of 43% of the transcripts and may therefore still be on the border to be considered an efficiently edited site. The other substrates with an editing efficiency of 7–45% have conserved sequence at the edited site and the ECS but no flanking conserved sequence. These results indicate that efficiently edited sites in general are flanked by stable stem structures that could function as EIEs to recruit ADAR, while sites with low editing efficiency rely on inefficient enzyme recruitment reflected in a lower level of editing.

### Editing in Kv1.1 can be increased by an EIE

**Fig. 5:**
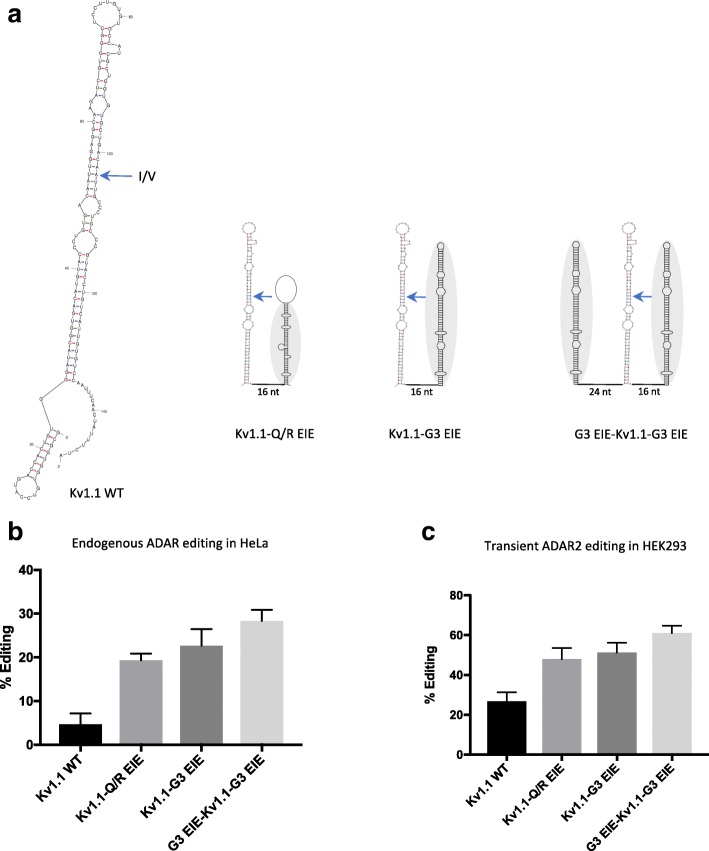
Editing at the I/V site of Kv1.1 is induced by an EIE. **a** *Left*: mfold structure prediction of the Kv1.1 transcript in the vicinity of the I/V site. The *blue arrow* indicates the I/V site. *Right*: the three Kv1.1 I/V editing reporter constructs illustrating the insertion of the EIE from GluA2 (kv1.1-Q/R EIE), the insertion of the EIE from Gabra-3 (Kv1.1-G3 EIE), and the insertion of the Gabra-3 EIE both upstream and downstream of the Kv1.1 stem loop. **b** Quantification of editing efficiency at the I/V site from the different Kv1.1 constructs transfected into HeLa cells, as indicated. **c** Quantification of editing efficiency at the I/V site from the different Kv1.1 constructs co-transfected with ADAR2 in HEK 293 cells. The mean value of the ratio between the A and G peak heights from three individual experiments was calculated as percentage of editing. *Error bars* are standard deviation

The mammalian potassium channel transcript Kv_1.1_ or KCNA1 is edited by ADAR2 at one site, creating an amino acid change in the translated protein (I/V) [39]. This transcript is intronless and the small hairpin creating the editing substrate consists entirely of exon sequence. On average, Kv_1.1_ is edited in no more than 25% of the transcripts in the adult mouse brain [31]. As mentioned above, conserved dsRNA structures flanking the edited site in this substrate could not be found. To investigate if editing at the I/V site could be induced by the addition of an EIE, we made an editing reporter expressing the RNA stem loop structure known to be required for editing at the I/V site in Kv_1.1_ (Kv1.1 WT) (Fig. 5a). Only 5% of the transcripts expressed from this reporter were edited by the endogenous ADAR2 (Fig. 5b). Strikingly, when the EIE from GluA2 was inserted downstream of the Kv_1.1_ stem loop (Kv1.1-Q/R EIE), editing increased to 20%. A similar increase in editing efficiency could be observed when the EIE from the Gabra-3 transcript was placed downstream of the I/V stem loop, and an increase to about 30% editing when the Gabra-3 EIE was inserted both upstream and downstream of the Kv_1.1_ substrate (G3 EIE.Kv1.1-G3 EIE) (Fig. 5b). Transient co-transfection of ADAR2 with the different reporters in HEK293 cells showed a similar result (Fig. 5c). Here the wild-type sequence of Kv_1.1_ was edited to a similar level as in vivo, 25%, which could be increased to 60% with the addition of two Gabra-3 inducer elements (G3 EIE.Kv1.1-G3 EIE). These results indicate two things: i) editing at the I/V site in Kv_1.1_ is low due to the limited capability of the specific substrate to attract the ADAR2 enzyme; and ii) editing of the Kv_1.1_ RNA can be induced by the addition of stem structures in *cis*, ideal for ADAR recruitment.

### Editing within non-coding RNA is also induced by EIEs

**Fig. 6:**
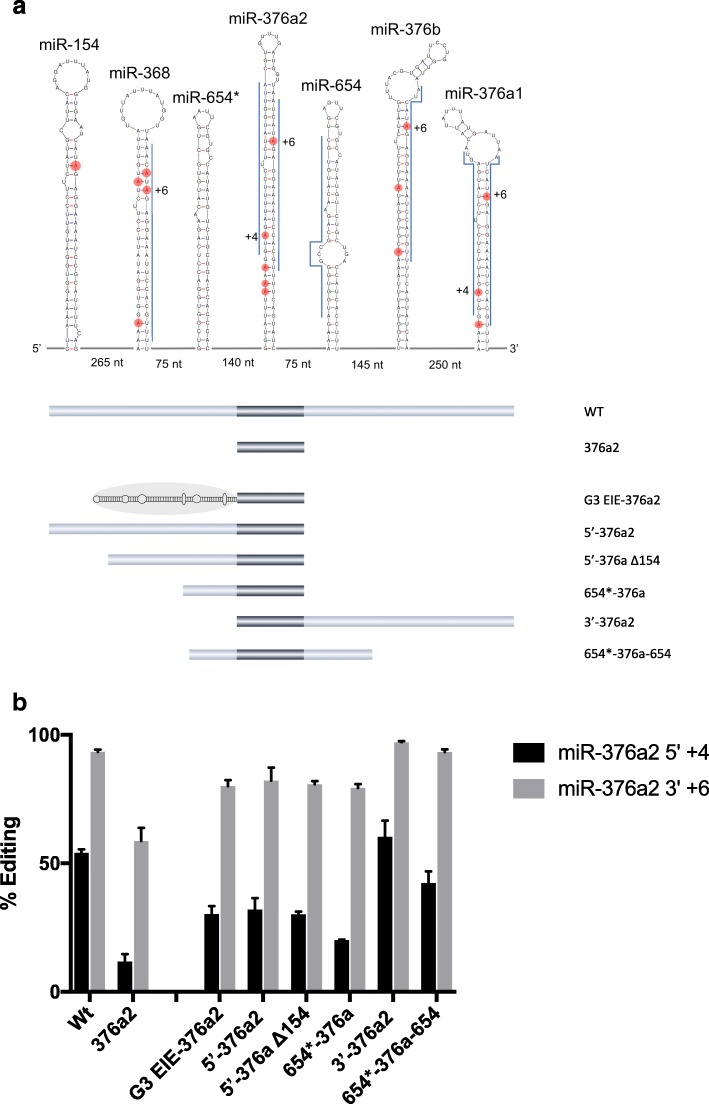
Structural requirements for efficient editing at the 5′ +4 site and 3′ +6 sites of the pri-miR-376a2 transcript. **a** *Top*: the miR-376 cluster constructs. Edited adenosines in the pri-miRNA stem loops are indicated with *red dots* and efficiently edited adenosines are shown with numbers indicating their position in the corresponding mature miRNAs. Mature miRNA sequences are indicated with *blue lines. Below*: sequences of different pre-miR constructs from the miR-376 cluster. *WT* indicates the full length of the cluster shown above. **b** Quantification of editing efficiency at the 5′ +4 site and 3′ +6 sites of the pri-miR-376a2 in the different constructs transfected into HeLa cells. The mean value of the ratio between the A and G peak heights from three individual experiments was calculated as percentage of editing. *Error bars* are standard deviation

In the substrates analyzed so far in this study, the specific editing sites are located within coding sequence of mRNAs, giving rise to amino acid changes in the translated proteins. To determine if editing can be induced by EIEs also in non-coding sequences we analyzed editing within the human miR-376 cluster. This cluster has been shown to be highly edited in the mature sequence of several pri-miRNAs [40]. A miRNA/editing reporter construct was made consisting of seven pri-miRNAs in the most highly edited region of the cluster (Fig. 6a). This part of the cluster is expressed as one continuous transcript (data not shown), indicating that it can be targeted for simultaneous co-transcriptional editing. When transfected into HeLa cells, the most efficient editing was found at the +6 site of miR-376a2-3′ where over 90% of the transcripts were edited (Fig. 6b). We therefore chose to focus on how editing at the +6 site was influenced by the other stem loops. When expressed in HeLa cells as a single stem loop, editing at +6 of pri-miR-376a2 was dramatically decreased to about 60%. The effect was even more dramatic at the 4+ site on the other strand of the pri-miRNA, miR-376a2-5′, where editing decreased from 55 to 13%, when expressed without the other stem loops in the vicinity (Fig. 6b). To determine if editing efficiency could be rescued by an EIE, we fused the EIE of Gabra-3 with pri-miR-376a2 in a reporter. Indeed, insertion of an upstream EIE rescued editing at the +6 site from 60 to 80% and at the +4 site from 13 to 30%. This result indicates that flanking sequence helps induce editing of pri-miR-376a2, even though it is not required for catalysis, and that this sequence most likely consists of a stem loop structure acting as an EIE.

To investigate if one or several stem loop structures in the vicinity of pri-miR-376a2 act as EIEs, we did consecutive deletions of the stems in the cluster (Fig. 6a). Deleting all stems 3′ of miR-376a2 lowered editing to some extent from over 90 to 80% (Fig. 6b). Furthermore, pri-miR-654*, immediately upstream, was sufficient to provide efficient editing of approximately 80% at the +6 site of miR-376a2. Interestingly, miR-654* lacks known editing sites and it is still unknown if it is processed into a mature miRNA in vivo. However, editing efficiency at +6 and +4 was unaffected by a deletion of all stems upstream of miR-376a2 (3′ 376a2), indicating that it is the stem(s) 3′ of these editing sites that are most important for editing induction. A construct with the two stems immediately upstream and downstream of miR-376a2 (654* + 376a2 + 654) showed rescue of 40% editing at the +4 site and over 90% at the +6 site. This result indicates that it is the stems immediately upstream and downstream of miR-376a2 that function as EIEs, with the most prominent one located downstream of the specific editing sites. In conclusion, specific editing sites located in non-coding sequences such as miRNAs can depend on inducer elements for efficient editing and these EIEs may consist of other pri-miRNAs.

## Discussion

**Fig. 7:**
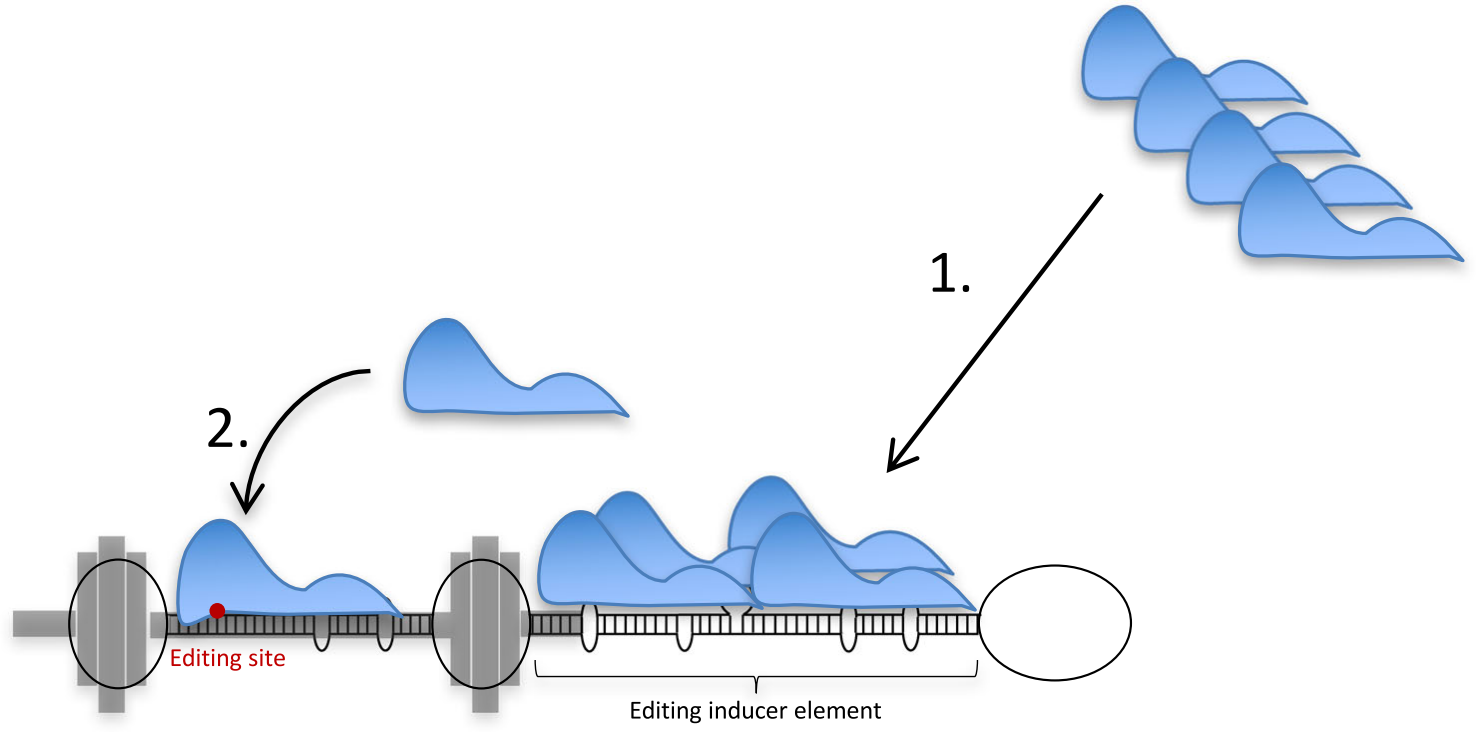
A model for efficient site selective A-to-I editing using an editing inducer element (EIE). The process of efficient editing occurs as two consecutive events: *1*) ADAR (in *blue*) recognizes a longer intronic stem by a non-specific interaction; *2*) when the ADAR enzymes have been recruited, the catalytic domain of the protein interacts with a specific site, ideal for catalysis, situated in a shorter stem limited by a barbell-like structure (in *grey*). The site of selective editing is indicated in *red*

We have previously shown that EIEs consisting of long stem loop structures can attract ADAR1 and ADAR2 to facilitate catalysis within adjacent shorter stem structures [28, 30]. In the present report, we show that EIEs are used as a general mechanism to increase editing efficiency at specific sites in both coding and non-coding RNA. Furthermore, the EIE is required to be detached from the specific editing site as a separate stem. Our present data suggest that the editing enzyme utilizes large internal loops as helix ends to increase both selectivity and efficiency of editing at specific sites within coding sequence. We propose that the ADAR enzymes recognize their substrates in two separate events: first they are attracted to a duplex structure that is ideal for protein binding but not necessarily for editing efficiency or specificity, then to an adjacent shorter duplex holding the specific editing site (Fig. 7). The first event attracts the enzyme to a longer double-stranded region, which may be ideal for binding but not catalysis. This will increase the local concentration of the enzyme at the substrate, inducing editing at a second site with a lower binding affinity. The second site is more specific since it requires a certain nucleotide sequence, ideal for efficient catalysis but also with restraints on non-specific editing.

By in vitro analysis it has previously been shown that dsRNA flanked by internal loops larger than 6 nt are identified as helix ends by the ADAR1 enzyme, while smaller loops of up to 4 nt act as part of the helix [41]. Limiting the length of the helix by larger internal loops, forming a “barbell” structure, forces ADAR into the right position for efficient site-specific editing and removes the risk of receiving non-specific adjacent editing. In our model, highly efficient site selective A-to-I editing within coding sequence commonly requires adjacent but separate double-stranded RNA structures that attract the editing enzyme to the substrate and thereby increase the local concentration of the protein. To test this theory, we removed the internal loop of 30 nt separating the stem including the Q/R site in the GluA2 transcript from the longer intronic stem downstream. Removal of the internal loop led to more promiscuous editing at several sites also within coding sequences (Fig. 3). Surprisingly, it also led to a dramatic decrease in editing by 50% at the specific Q/R site. This result reveals that separate stem structures are required for recruitment and efficient catalysis.

Like other dsRNA binding enzymes such as PKR, Staufen, and Drosha, the ADAR enzymes are thought to interact promiscuously with dsRNA via their dsRNA binding domains, as they recognize the minor groove in the sugar-phosphate backbone of the RNA without any direct contact with the nucleotide bases [14, 42]. Hence, the ADAR editing enzymes have the potential to bind any base paired structure of RNA. However, the two dsRBDs in ADAR2 have previously been shown to selectively bind to the stem at the Q/R site and also contribute to editing efficiency in vitro [43]. It is therefore likely that the ADAR2 interaction at the Q/R site is different from the interaction with the downstream stem, which is also edited but with a lower efficiency. To determine the role of the dsRBDs in editing efficiency and specificity in vivo, we analyzed the editing pattern of an ADAR2 enzyme with mutated dsRBDs on the GluA2 substrate. This mutant is unable to bind RNA via its dsRBDs. Similar to the in vitro analysis, editing efficiency was severely affected by the mutations in the dsRBDs. Furthermore, no editing was detected in the absence of the EIE, indicating that this element is important for editing efficiency also in the absence of the dsRBDs (data not shown). However, little difference in sites selected for editing by the dsRBD mutant could be detected compared to editing by wild-type ADAR2. Nevertheless, the editing level of the +60 site is comparatively high in the dsRBD mutant, indicating that this is a primary docking site for ADAR2. In summary, this indicates that the dsRBDs and deamination domain both contribute to recruitment and catalysis and that the dsRBDs contribute to a minor extent to site selectivity.

Co-crystallization of the ADAR2 deaminase domain together with an RNA substrate revealed that the deamination domain also specifically interacts with the RNA helix structure, covering 20 bp [16]. During deamination, the catalytic site of the ADAR enzymes flips the adenosine out of the helix in order to make it accessible by the active site [44]. Specific residues of the ADAR2 protein are in contact with the RNA during deamination, which also offers an explanation for the difference in substrate selectivity between ADAR1 and ADAR2 [16]. Furthermore, the co-crystal structure also reveals the preference for the 5′ and 3′ nearest neighbor of the edited adenosine, explained by interactions with the catalytic ADAR2 domain during the base-flipping reaction. Limiting the positioning of the ADAR protein, either by a short stem loop or by internal loops in a longer stem structure, facilitates specificity and efficiency but is not necessarily the most efficient way of attracting the enzyme to the substrate.

In mouse and human, editing has been shown to be regulated within both non-coding RNA and gene transcripts during development [31, 45–47]. Editing rates can be divided into three groups: stable high, developmentally increasing, and stable low. The Q/R site in GluA2 belongs to the first category while the Q/R site in GluK2 and the I/M site in the Gabra-3 transcript increase during development. In the adult brain the Q/R site of GluA2 is edited in nearly 100% of all transcripts, while editing of GluK2 at the Q/R site reaches over 80% editing in the adult mouse brain and Gabra-3 over 90% [31]. We define both of these categories as high efficiency sites and found editing inducer elements adjacent to all the efficiently edited sites within coding regions analyzed (Table 1). Efficient enzyme recruitment may therefore explain their high efficiency of editing in adult tissue and indicates that the presence of EIEs is a general mechanism used to increase the efficiency of editing at specific sites. Editing at the Q/R site in GluA2 is, however, highly efficient also in the embryonic brain where GluK2 and Gabra-3 editing is barely detected. The high level of GluA2 Q/R editing in the embryo might be explained by a higher affinity of the ADAR2 enzyme to this site than to other sites of editing. Recent data from our laboratory indicate that concentrations of the ADAR2 enzyme are lower in the nucleus of premature embryonic neurons than mature neurons [48]. A-to-I editing is a nuclear event and is therefore dependent on the level of nuclear ADAR. By transient transfection of ADAR2 we show that an extremely low level of ADAR2 is required for efficient editing at the Q/R site of GluA2 but only in the presence of the EIE. Our result gives a plausible explanation to the enigma of how GluA2 Q/R editing can be edited to 100% while other sites are unedited in the embryonic brain.

Interestingly, we also show that EIEs can induce editing in non-coding RNA. We and others have shown that several miRNAs in one specific cluster (miR379-410) in mouse are subjected to A-to-I editing in their target recognition sequence [40, 49]. Most editing events in these miRNAs are also conserved between human and mouse. In human, the homologous miR-376 cluster consists of more than 40 miRNAs and it has been suggested that the entire cluster functions as a tumor suppressor locus. In a reporter construct, consisting of seven pri-miRNAs from this cluster, we analyzed if editing efficiency was influenced by adjacent pri-miRNA stem structures. Specifically, we analyzed the +6 site of miR-376a2-3', as it was the most efficiently edited site in the cluster. Indeed, we found that efficient editing of miR-376a2 was dependent on two stem structures of pri-miRNAs immediately upstream and downstream. Our results show that editing within miRNA sequences can be induced by other pri-miRNAs, functioning as EIEs. This may also explain why editing within miRNAs is a relatively rare event when expressed as singular pri-miRNAs and not in clusters.

## Conclusions

An increasing amount of transcriptomics data provide evidence of A-to-I RNA editing playing an important role in specific tissues in response to external stimuli or stress as well as in developmental regulation and immunity. In order to verify these editing events, we need to know the structural requirements for substrate recognition. Revealing the mechanism and components required for efficient editing will contribute to an increased understanding of the variations in levels of A-to-I RNA modification. By understanding how a substrate is selected for editing we will also be able to discover new sites of editing as well as understand the cause of aberrant editing related to cancer progression as well as immunological and neurological disorders. Double-stranded RNA plays a key role in many biological functions in cells, including RNA interference, anti-viral immunity, and mRNA transport. Responsible for recognizing dsRNA are a class of dsRNA binding proteins (dsRBPs), including ADAR. Our novel way of explaining substrate selectivity and efficiency may therefore also relate to other dsRBPs, such as Staufen 1 and Drosha, for which little is known about the molecular mechanism underlying substrate recognition.

## Methods

### Plasmids and substrate mutagenesis

The ADAR2 expression vector has been previously described [27, 50]. The ADAR1 expression vector pCS DRADA-FLIS6 [51] was a kind gift from Mary O’Conell. The mouse GluA2 Q/R(GA2Q/R), Grik2 Q/R(GK2Q/R), and human miR-376 cluster editing reporter constructs were generated by polymerase chain reaction (PCR) amplification from genomic DNA and cloned into pcDNA3 FLAG. Primer sequences were as follows: GluA2 forward (FW) 5′-ctggatgtgcattgtgtttg-3′, reverse (RE) 5′-gaccctgtaggaaaaatctaacctc-3; GluK2 FW 5′-tgttggatagaatcttctcactgc-3′, RE 5′-gcacatgttttcaatgttagca-3; miR376 cluster FW 5′- catgtttgcgtttgtgctct-3′, RE 5′-ctccgaggttttcaaagcag-3′; 376a2 FW 5′-tcctctgtgctatgttacttttgtg-3′, RE 5′-ctgatggtggcttcagtcc-3′; 5′-376a2 FW 5′-catgtttgcgtttgtgctct-3′, RE 5′- ctgatggtggcttcagtcc-3′; 3′-376a2 FW 5′-tcctctgtgctatgttacttttgtg-3′, RE 5′- ctccgaggttttcaaagcag-3′; 654*-376a-654 FW 5′-gcttggaaacattcctggac-3′, RE 5′-cgttttcagtcccgtagcat-3′. The deletion mutants GA2Q/R ΔEIE, GK2Q/R-ΔEIE, and GA2Q/R-Δloop were generated from GA2Q/R and GK2Q/R. ADAR2-EAA, ADAR-E488Q, and ADAR2-EAA-E488Q were generated from ADAR2 using QuikChange II™ site-directed mutagenesis (Stratagene/Agilent Technologies) following the manufacturer’s instructions. 5′-376a Δ154 and 654*-376a constructs were generated by deletions of miR-154 and miR-368* using QuikChange II™ site-directed mutagenesis (Stratagene/Agilent Technologies) following the manufacturer’s instructions. The GA2Q/R-US EIE, GA2Q/R-US G3, Kv1.1, Kv1.1-Q/R EIE, Kv1.-G3 EIE, and G3 EIE-Kv1.1-G3 EIE sequences were synthetically designed (IDT) and cloned into the EcoRV restriction enzyme site of pcDNA3 FLAG using NEBuilder HiFi DNA Assembly (New England Biolabs) according to the manufacturer’s instructions. The Gabra-3 editing reporter construct G3 I/M (pGARα3-I/M) and the deletion mutant G3-ΔEIE (Gabra3-Δ149) have been previously described [28]. To generate the G3 I/M-DS GA2 EIE construct the GluA2 Q/R EIE was amplified by PCR and cloned into the Gabra-3 construct at the position of the Gabra-3 EIE. The G3 EIE-376a2 construct was generated by PCR amplification and cloning into the 376a2 construct as described previously [28].

All plasmids and mutants were verified by Sanger sequencing (Eurofins MWG operon).

### Transfections

GluA2 reporter constructs GA2Q/R, GA2Q/R ΔEIE, GA2Q/R-US EIE, GA2Q/R-US G3 EIE, and GA2Q/R-Δloop and Kv1.1 reporter constructs Kv1.1, Kv1.1-Q/R EIE, Kv1.-G3 EIE, and G3 EIE-Kv1.1-G3 EIE (0.75 μg) were co-transfected with the ADAR2 (100 ng) expression vector into HEK293 cells and grown in 12-well plates. For endogenous editing, the GluA2, GluK2, and Kv1.1 reporter constructs (100 ng) were transfected into HeLa cells grown in 12-well plates. The Gabra3 reporter constructs G3I/M, G3I/M-ΔEIE, G3I/M-DS, and GA2 EIE (0.75 μg) were co-transfected with ADAR1 or ADAR2 (1.25 μg) expression vectors into HEK293 cells and grown in 12-well plates. For endogenous editing, the Gabra3 (2 μg) reporter constructs were transfected into HeLa cells grown in 12-well plates.

In the ADAR2 titration experiments a fixed amount of 0.75 μg reporter constructs GA2Q/R or GA2Q/R ΔEIE was co-transfected with varying amounts of ADAR2 expression vector (1.25, 0.75, 0.5, 0.25, 0.05, or 0 μg) into HEK293 cells and grown for 48 h in 12-well plates. For the ADAR mutant experiments a fixed amount of 0.75 μg reporter constructs GAQ/R and GA2Q/R-Δloop was co-transfected with 0.8 μg ADAR mutant expression vector into HEK293 cells and grown in 12-well plates for 48 h. The ADAR protein level was controlled by western blot analysis. For the miR-376a2 editing reporter constructs, 2 μg of the constructs were transfected into HeLa cells grown in 12-well plates. LIPOFECTAMINE™ 2000 (Invitrogen) was used in all transfections. The transfection efficiency was comparable between separate experiments. As controls, co-transfections with an empty expression vector instead of ADAR2 were done for each experiment. RNA was isolated 48 h (HEK293 and miRNA constructs in HeLa) and 72 h (HeLa) after transfection using GenElute™ mammalian total RNA isolation (Sigma), and treated with DNase-1 Amplification Grade (Sigma). cDNA was generated using random hexamer deoxyoligonucleotides and SuperscriptII RT (Invitrogen). Negative control reactions without reverse transcriptase were performed in all RT-PCR experiments to exclude genomic DNA contamination. The following PCR was made using Taq (Invitrogen). Primers used for the PCR reactions were as follows: for GA2Q/R and GA2Q/R ΔEIE/Δloop reporters, FW 5′-cctggtcagcagatttagcc-3′, RE 5′-tgctagagctcgctgatcag-3′; for GA2Q/R-US EIE, FW 5′-ttgatcatgtgtttccctggt-3′, RE 5′-aaacacggtacccctccaag-3′; for GA2Q/R-US G3 EIE, FW 5′-aggaactcagcagggctatg-3′, RE 5′-gagaatatgcagcaaaaacacg-3′; for G3I/M, G3I/M-ΔEIE, and G3I/M-DS GA2 EIE, FW 5′-ggtgtcaccactgttctcacc-3′, RE 5′-gctgtggatgtaataagactcc-3; for GK2Q/R and GK2Q/R ΔEIE, FW 5′-gatagaatcttctcactgctat-3′, RE 5′-caaattgagacaggaaacagg-3′; for Kv1.1, Kv1.1-Q/R EIE, Kv1.-G3 EIE, and G3 EIE-Kv1.1-G3 EIE, FW 5′-aactttgtgcattttaggtc-3′, RE 5′-aaccttctgcattttatagcc-3′; for miR-367a2, FW 5′-taatacgactcactataggg-3′, RE 5′-ctgatggtggcttcagtcc-3′.

### Calculation of editing frequency

To evaluate the level of edited transcripts, RNA from at least three independent experiments was sequenced. Editing was determined by measuring the ratio between the A and the G peak height in individual chromatograms using FinchTV. The percentage of editing was calculated as the peak height of G/(A + G) × 100.

### Prediction of RNA secondary structure

RNA secondary structure predictions were made through Mfold [37] and ViennaRNA Package 2.0 [38]. All secondary structures mentioned were observed by algorithms.

## Additional files

Additional file 1: Figure S1. Quantification of editing efficiency at the Q/R sit from the different GA2Q/R reporters cotransfected with ADAR2 in HEK 293 cells. The mean value of the ratio between the A and G peak heights from three individual experiments are calculated as percentage of editing. Error bars are standard deviation. The value of GA2Q/R-ΔEIE was significantly different to the values of all the other reporters, the values of the GA2Q/R-US EIE and GAQ/R-US G3 EIE where not significantly different to the WT GA2Q/R reporter (P=0.05 two tailed student’s ttest). **Figure S2.** Titration of ADAR2 co-transfected with GA2Q/R or GA2Q/R-ΔEIE. (**a**) Sequencing chromatograms of RT-PCR products from ADAR2 co-transfections with GA2Q/R or GA2Q/R-ΔEIE. In each experiment, transfection of the reporter constructs was constant (0.75μg), while the concentration of ADAR2 was titrated (0-1.25μg). (**b**) Quantification of the Q/R editing efficiency in GA2Q/R (dots) and GA2Q/R-ΔEIE (squares) reporters when co-transfected with titrated ADAR2. Three individual experiments were done for each concentration. The mean value of the ratio between the A and G peak heights was calculated as percentage of editing. Error bars are standard deviation. **Figure S3.** (**a**) Sites of editing and average % editing in the GluA2 reporter GA2Q/R cotransfected with the mutant ADAR2-E488Q expression vector in HEK293 cells. Below, sites of editing in the GluA2 reporter with the internal loop deleted (GA2Q/R-Δloop) co-transfected with ADAR2-E488Q in HEK293. The average value of the ratio between the A and G peak heights from two separate experiments was calculated as percentage editing. (**b**) Western blot showing expression levels of different transiently transfected ADAR2 expression vectors shown in A and Figure 3. EV equals transfection of empty vector as control. **Figure S4.** Predicted RNA secondary structure of the EIE in mouse GluA2 and GluK2. Differences in the human sequences are indicated by arrows and base changes in blue. Edited adenosines in the mouse sequence are shown in read. (PDF 3470 kb)

Additional file 2: Figure S5. Secondary structure predictions of the pre-mRNA sequence in the vicinity of the selectively edited sites listed in Table 1. (PDF 3234 kb)

## Abbreviations

ΔEIE: Deleted editing inducer element
ADAR: Adenosine deaminase that acts on RNA
A-to-I: Adenosine to inosine
bp: Base pair
dsRNA: Double-stranded RNA
EIE: Editing inducer element
I/M: Isoleucine to methionine
I/V: Isoleucine to valine
K/E: Lysine to glutamate
K/R: Lysine to arginine
miRNA: MicroRNA
PCR: Polymerase chain reaction
Q/R: Glutamine to arginine
R/G: Arginine to glycine
RT: Reverse transcription
S/G: Serine to glycine
UTR: Untranslated region
WT: Wild type.
