# Supplementary material for: Editing inducer elements increases A-to-I editing efficiency in the mammalian transcriptome
Source: Genome Biol. 2017 Oct 23;18:195. doi: 10.1186/s13059-017-1324-x (PMC5654063; doi:10.1186/s13059-017-1324-x)
Supplement: Supplementary file 1 — Quantification of editing efficiency at the Q/R sit from the different GA2Q/R reporters cotransfected with ADAR2 in HEK 293 cells. The mean value of the ratio between the A and G peak heights from three individual experiments are calculated as percentage of editing. Error bars are standard deviation. The value of GA2Q/R-ΔEIE was significantly different to the values of all the other reporters, the values of the GA2Q/R-US EIE and GAQ/R-US G3 EIE where not significantly different to the WT GA2Q/R reporter (P=0.05 two tailed student’s ttest). Figure S2. Titration of ADAR2 co-transfected with GA2Q/R or GA2Q/R-ΔEIE. (a) Sequencing chromatograms of RT-PCR products from ADAR2 co-transfections with GA2Q/R or GA2Q/R-ΔEIE. In each experiment, transfection of the reporter constructs was constant (0.75μg), while the concentration of ADAR2 was titrated (0-1.25μg). (b) Quantification of the Q/R editing efficiency in GA2Q/R (dots) and GA2Q/R-ΔEIE (squares) reporters when co-transfected with titrated ADAR2. Three individual experiments were done for each concentration. The mean value of the ratio between the A and G peak heights was calculated as percentage of editing. Error bars are standard deviation. Figure S3. (a) Sites of editing and average % editing in the GluA2 reporter GA2Q/R cotransfected with the mutant ADAR2-E488Q expression vector in HEK293 cells. Below, sites of editing in the GluA2 reporter with the internal loop deleted (GA2Q/R-Δloop) co-transfected with ADAR2-E488Q in HEK293. The average value of the ratio between the A and G peak heights from two separate experiments was calculated as percentage editing. (b) Western blot showing expression levels of different transiently transfected ADAR2 expression vectors shown in A and Figure 3. EV equals transfection of empty vector as control. Figure S4. Predicted RNA secondary structure of the EIE in mouse GluA2 and GluK2. Differences in the human sequences are indicated by arrows and base changes in blue. Edited adenosines [file 13059_2017_1324_MOESM1_ESM.pdf]

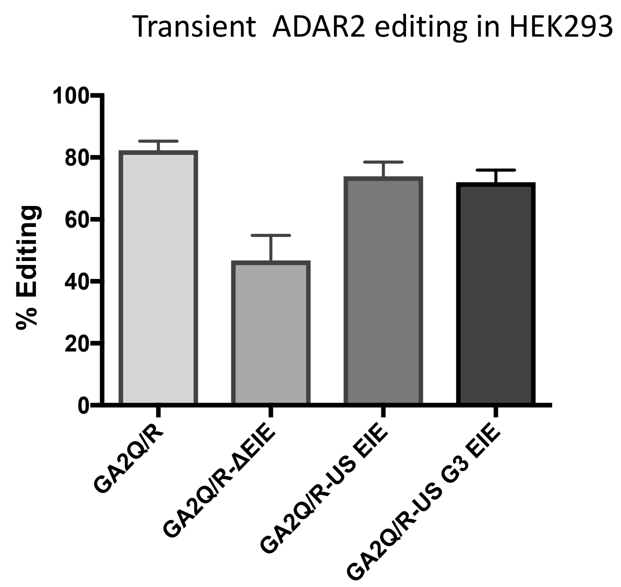

Figure S1

Quantification of editing efficiency at the Q/R sit from the different GA2Q/R reporters co-transfected with ADAR2 in HEK 293 cells. The mean value of the ratio between the A and G peak heights from three individual experiments are calculated as percentage of editing. Error bars are standard deviation. The value of GA2Q/R-ΔEIE was significantly different to the values of all the other reporters, the values of the GA2Q/R-US EIE and GAQ/R-US G3 EIE where not significantly different to the WT GA2Q/R reporter (*P=0.05* two tailed student's t-test).

a

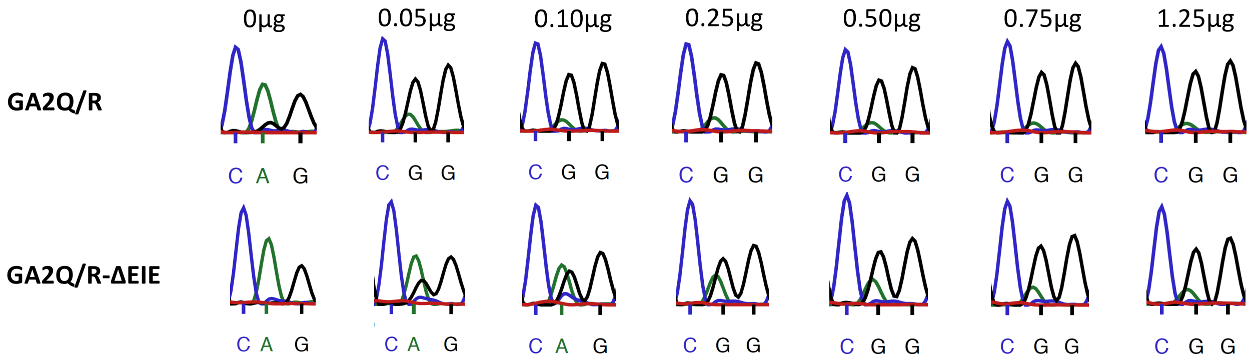

b

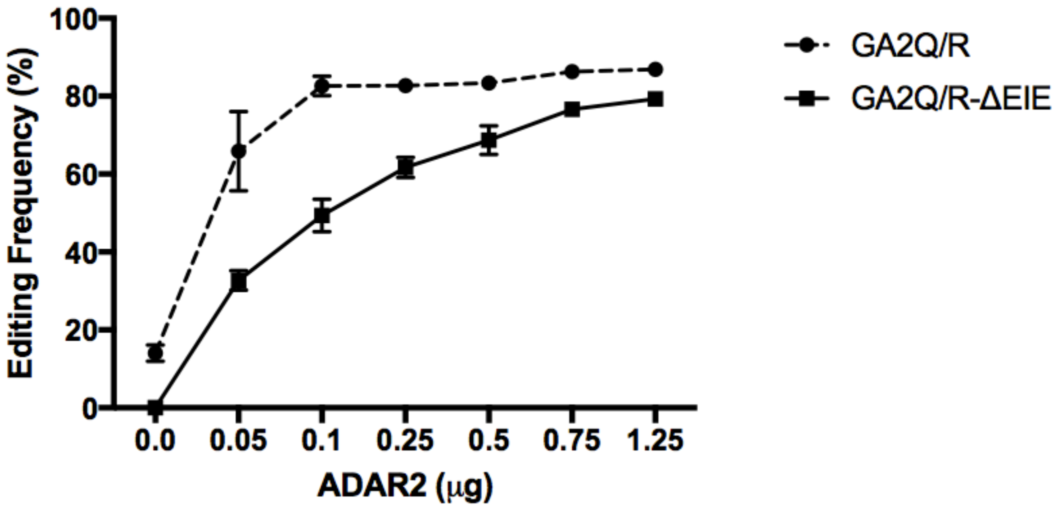

Figure S2
Titration of ADAR2 co-transfected with GA2Q/R or GA2Q/R-ΔEIE. **(a)** Sequencing chromatograms of RT-PCR products from ADAR2 co-transfections with GA2Q/R or GA2Q/R-ΔEIE. In each experiment, transfection of the reporter constructs was constant (0.75µg), while the concentration of ADAR2 was titrated (0-1.25µg). **(b)** Quantification of the Q/R editing efficiency in GA2Q/R (dots) and GA2Q/R-ΔEIE (squares) reporters when co-transfected with titrated ADAR2. Three individual experiments were done for each concentration. The mean value of the ratio between the A and G peak heights was calculated as percentage of editing. Error bars are standard deviation.

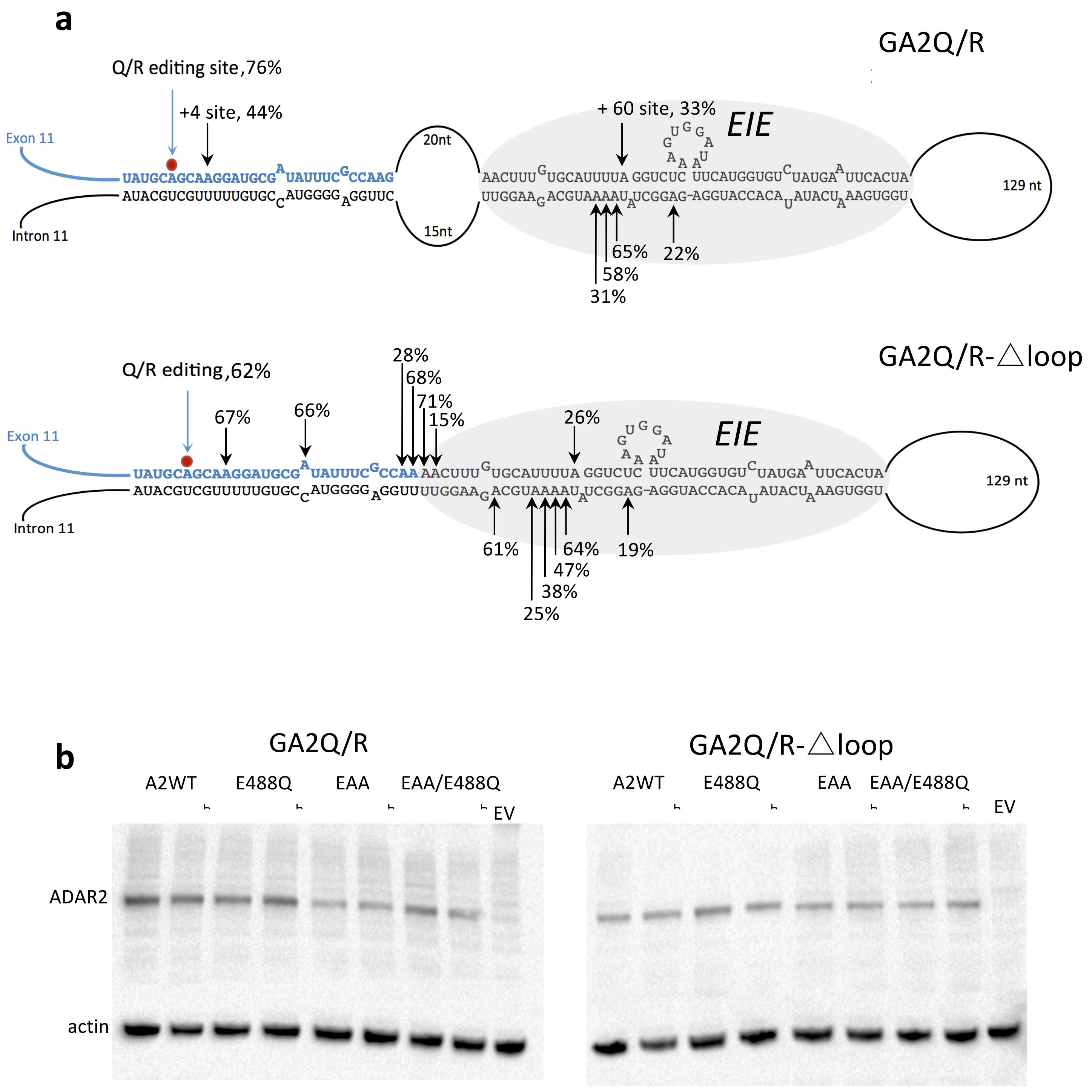

Figure S3
(A) Sites of editing and average % editing in the GluA2 reporter GA2Q/R co-transfected with the mutant ADAR2-E488Q expression vector in HEK293 cells. Below, sites of editing in the GluA2 reporter with the internal loop deleted (GA2Q/R-Δloop) co-transfected with ADAR2-E488Q in HEK293. The average value of the ratio between the A and G peak heights from two separate experiments was calculated as percentage editing. (B) Western blot showing expression levels of different transiently transfected ADAR2 expression vectors shown in A and Figure 3. EV equals transfection of empty vector as control.

mGluA2 EIE

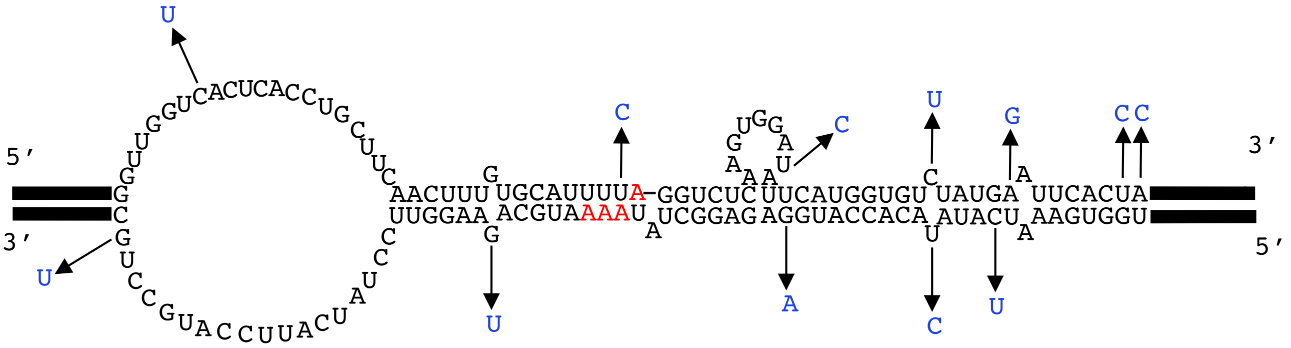

mGluK2 DSS EIE

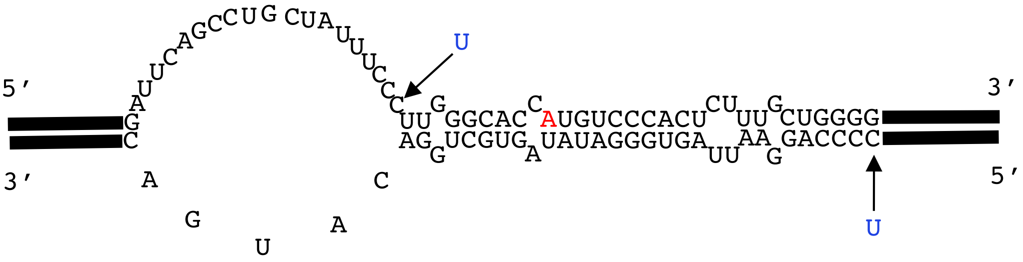

Figure S4
Predicted RNA secondary structure of the EIE in mouse GluA2 and GluK2. Differences in the human sequences are indicated by arrows and base changes in blue. Edited adenosines in the mouse sequence are shown in read.
