# Supplementary material for: Editing inducer elements increases A-to-I editing efficiency in the mammalian transcriptome
Source: Genome Biol. 2017 Oct 23;18:195. doi: 10.1186/s13059-017-1324-x (PMC5654063; doi:10.1186/s13059-017-1324-x)
Supplement: Supplementary file 2 — Secondary structure predictions of the pre-mRNA sequence in the vicinity of the selectively edited sites listed in Table 1. (PDF 3234 kb) [file 13059_2017_1324_MOESM2_ESM.pdf]

Figure S5. Secondary structure predictions of the pre-mRNA sequence in the vicinity of the selectively edited sites listed in Table 1. **(a)** GluA2 at the Q/R site. **(b)** Gabra3 at the I/M site. **(c)** GluA3 at the R/G site. **(d)** FLNB at the Q/R site. **(e)** Htrc2 at the I/V site. **(f)** GluK2 at the Q/R site. **(g)** ADAR2 at the +24 site. **(h)** Cyfip2 at the K/E site. **(i)** GluA2 at the R/G site. **(j)** GluK1 at the Q/R site. **(k)** IGFBP7 at the K/E site. **(l)** FLNA at the Q/R site. **(m)** Nova1 at the S/G site. **(n)** KCNA1 at the I/V site. **(o)** PLCH2 at the R/G site. **(p)** TMEM63B at the Q/R site. **(q)** CCNI at the R/G site. **(r)** Azin1 at the S/G site. **(s)** Copa at the I/V site. **(t)** GPATCH8 at the K/R site **(u)** NCSTN at the S/G site. **(v)** OSGEP at the I/M site. **(x)** BLCAP at the Y/C site.

Output of sir_graph (©)
mfold_util 4.7

Created Tue Jun 27 18:39:06 2017

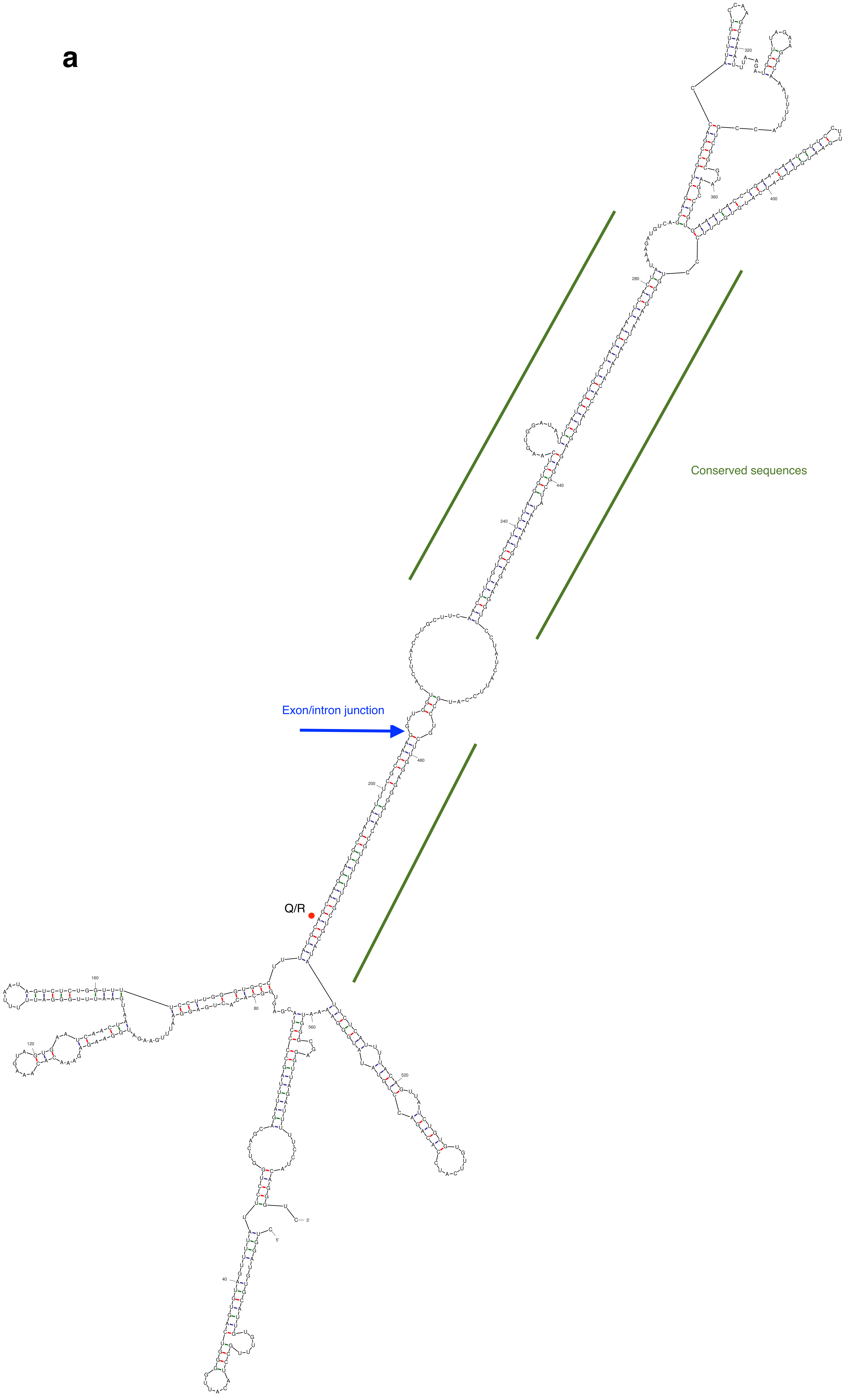

dG = -186.38 [Initially -197.20] Gria Q/R

b

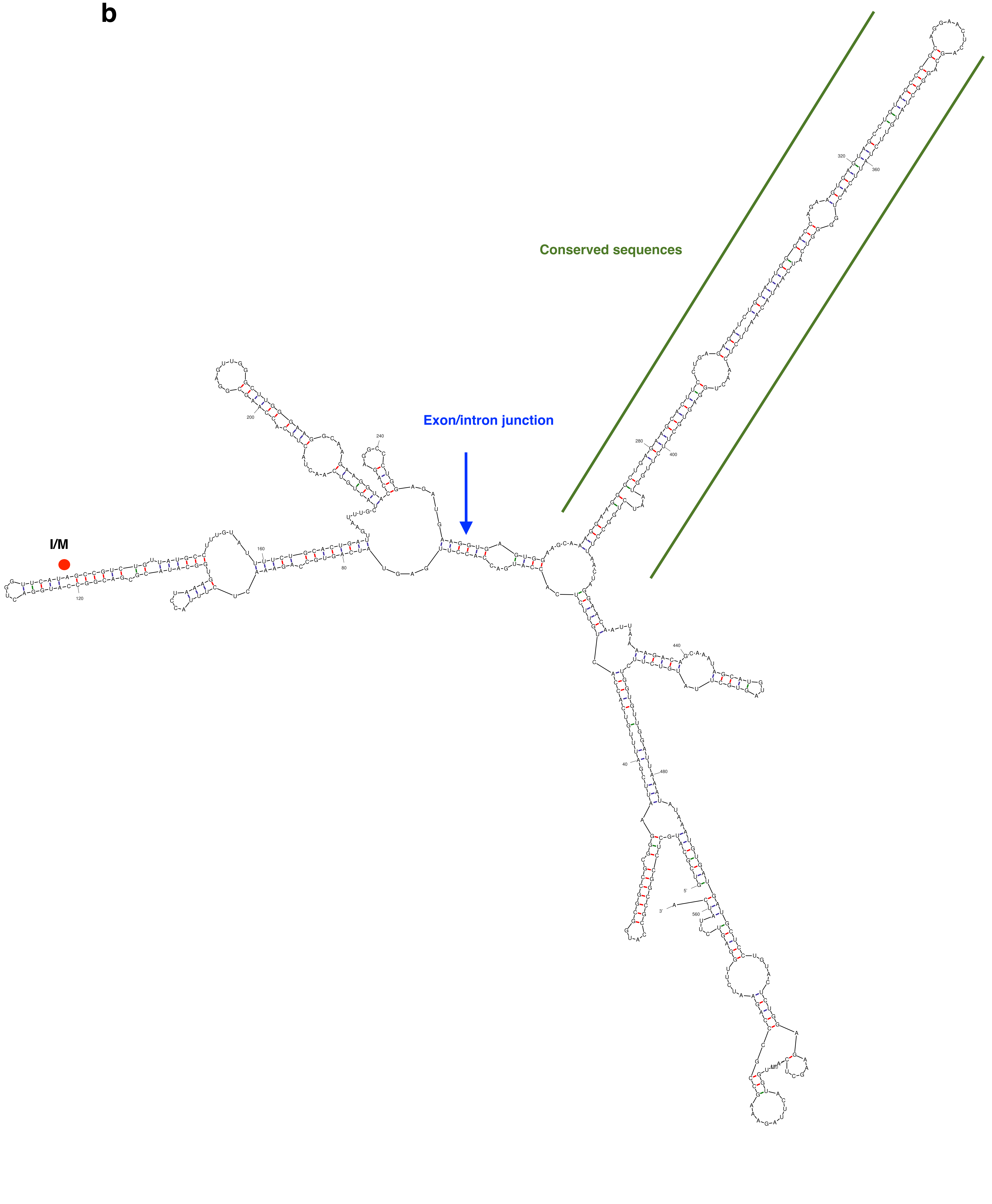

dG = -201.60 [Initially -212.40] Gabra3

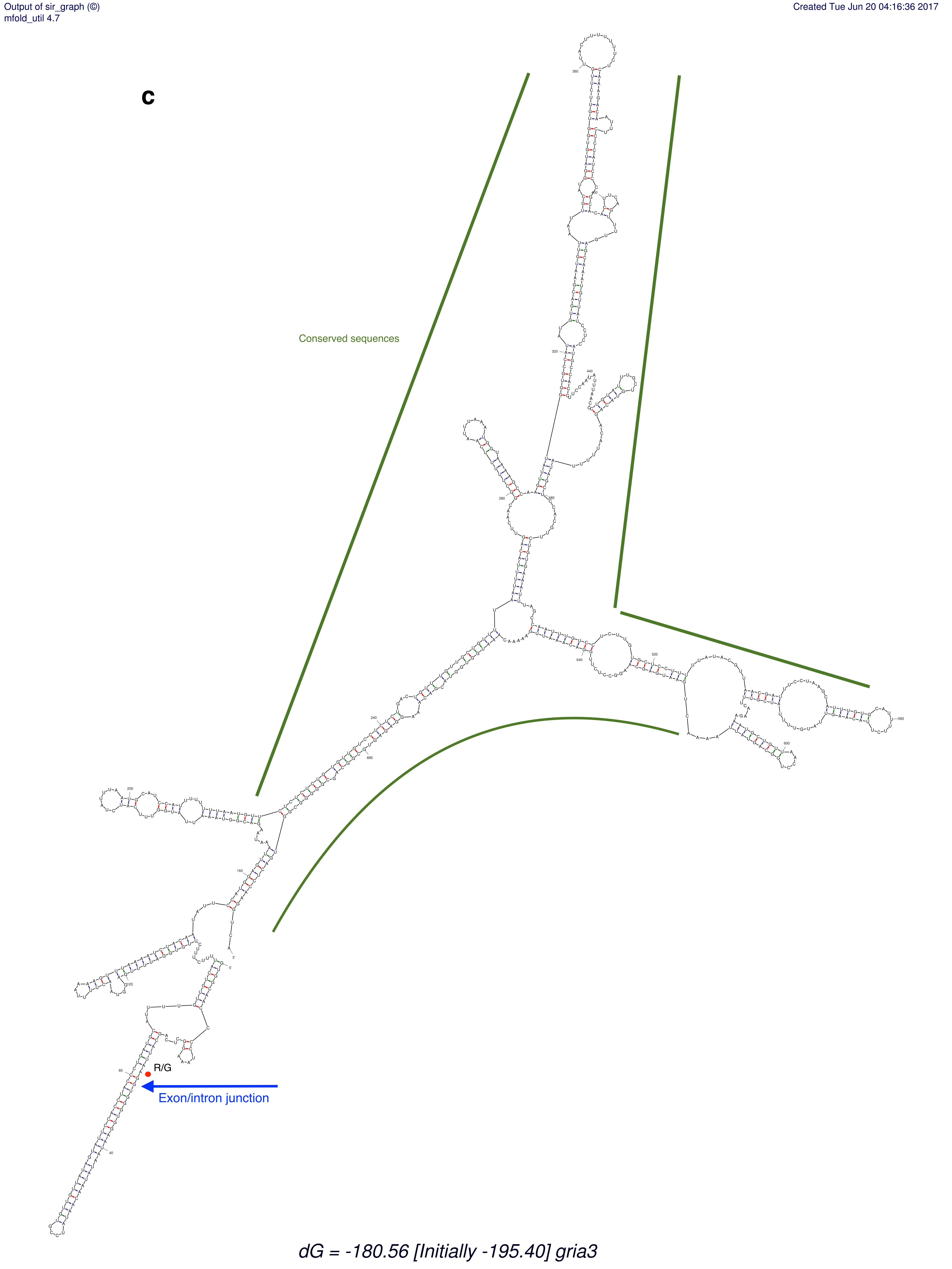
Output of sir_graph (©)
mfold_util 4.7
Created Tue Jun 20 04:16:36 2017
c
Conserved sequences
R/G
Exon/intron junction
dG = -180.56 [Initially -195.40] gria3

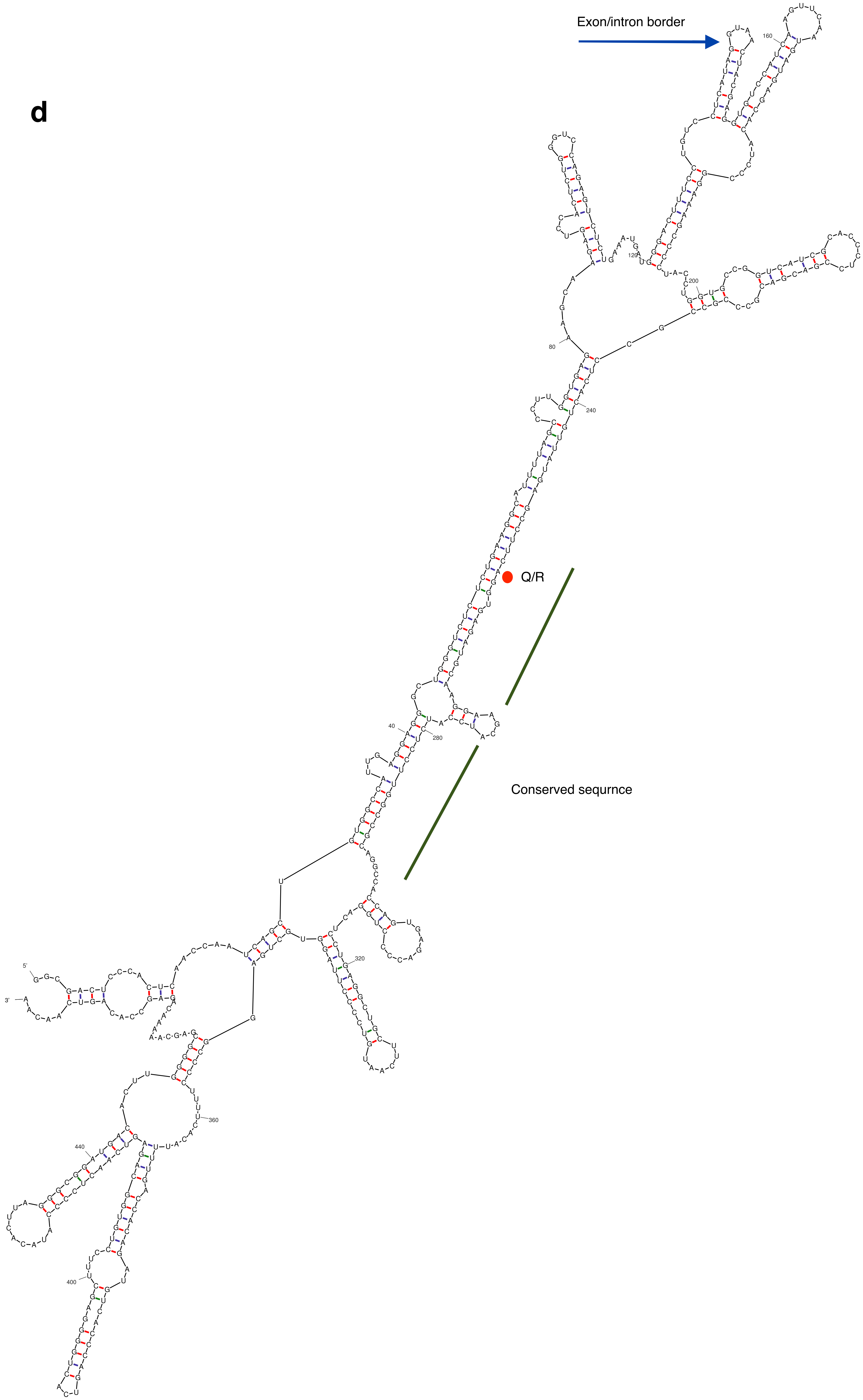

dG = -159.90 [Initially -178.00] 17Jun27-12-59-53

e

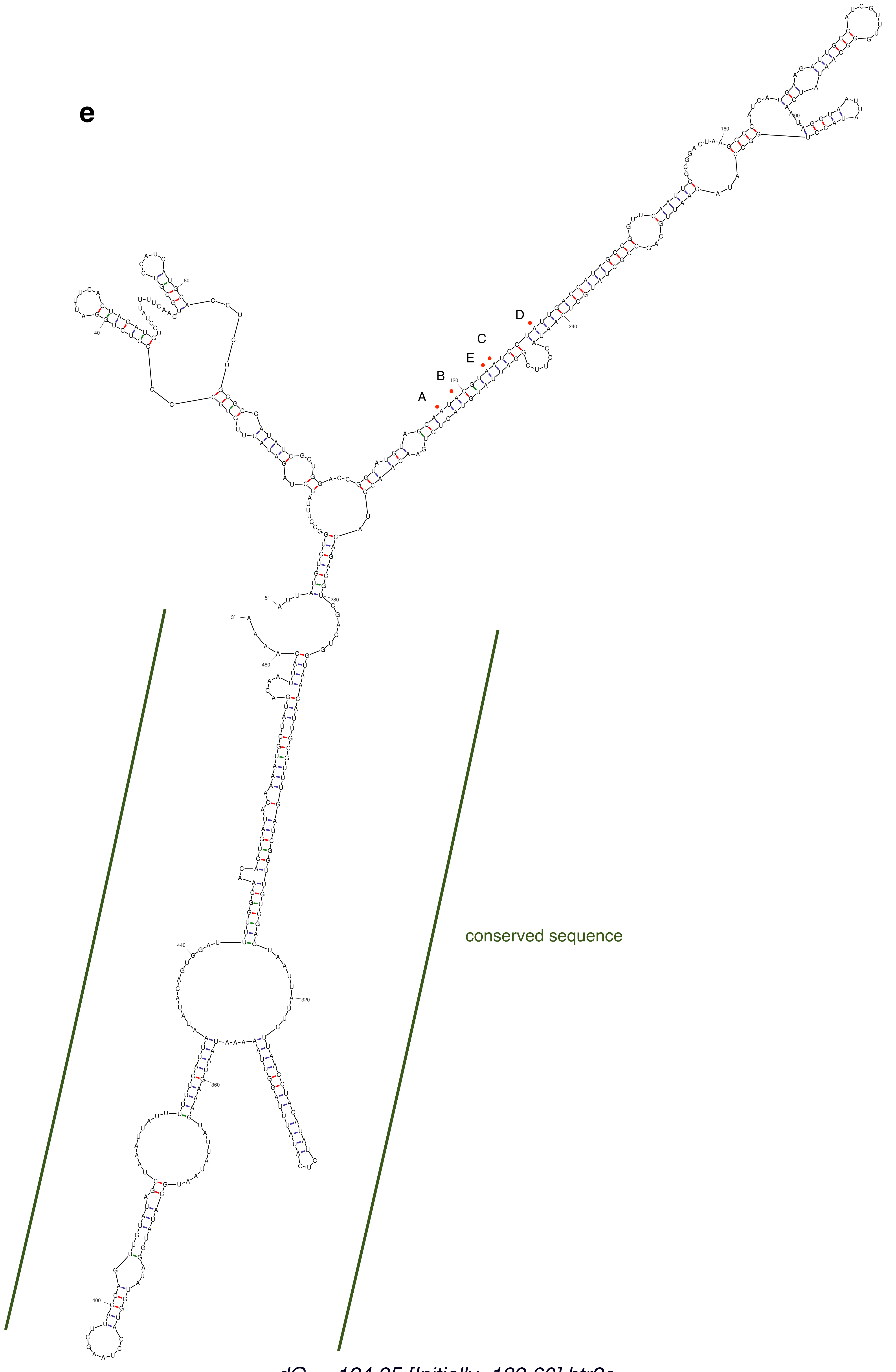

dG = -124.35 [Initially -139.60] htr2c

f

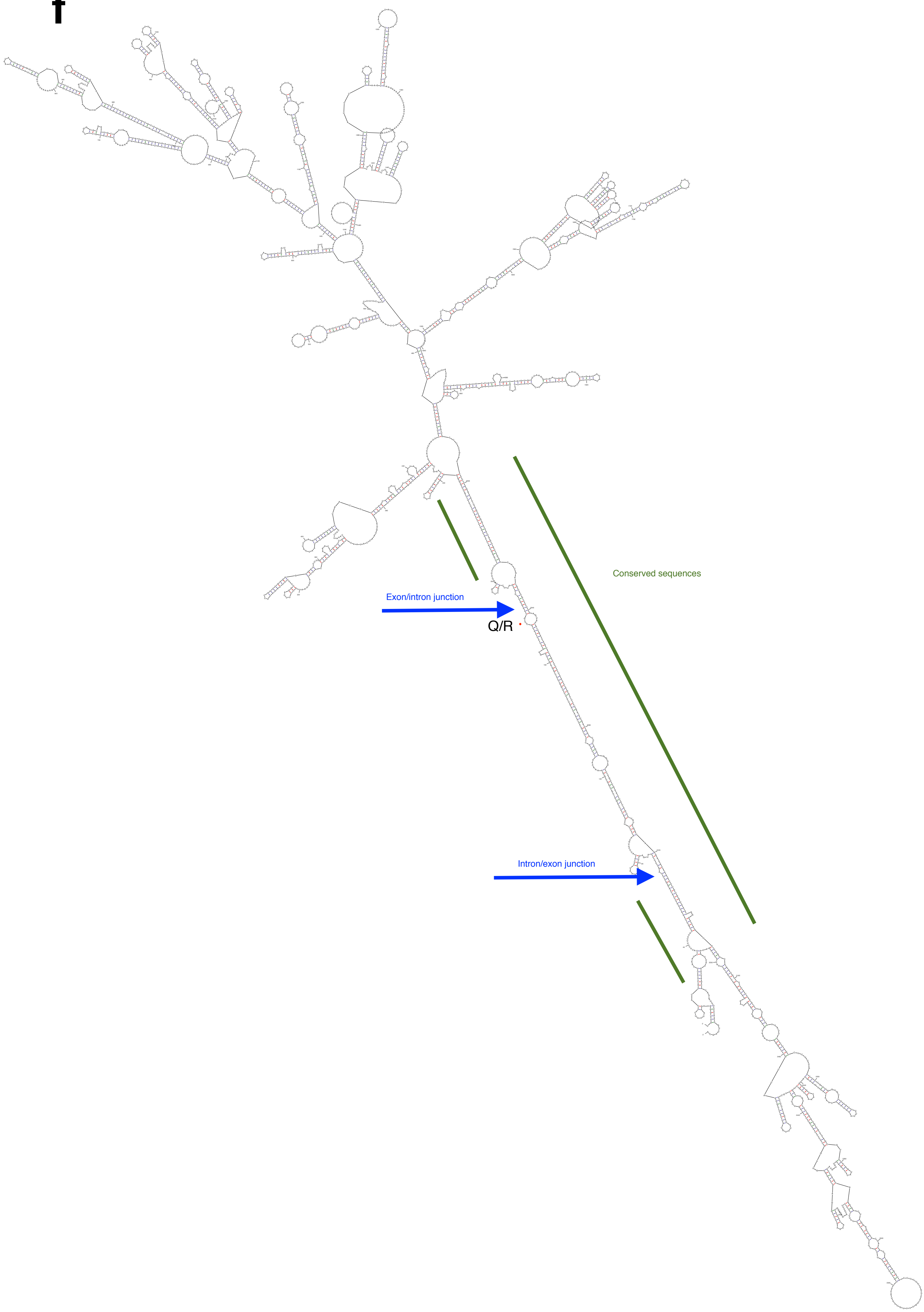

dG = -555.71 [Initially -630.00] Grik2 full length

Output of sir_graph (©)
mfold_util 4.7

Created Mon Jun 26 15:38:06 2017

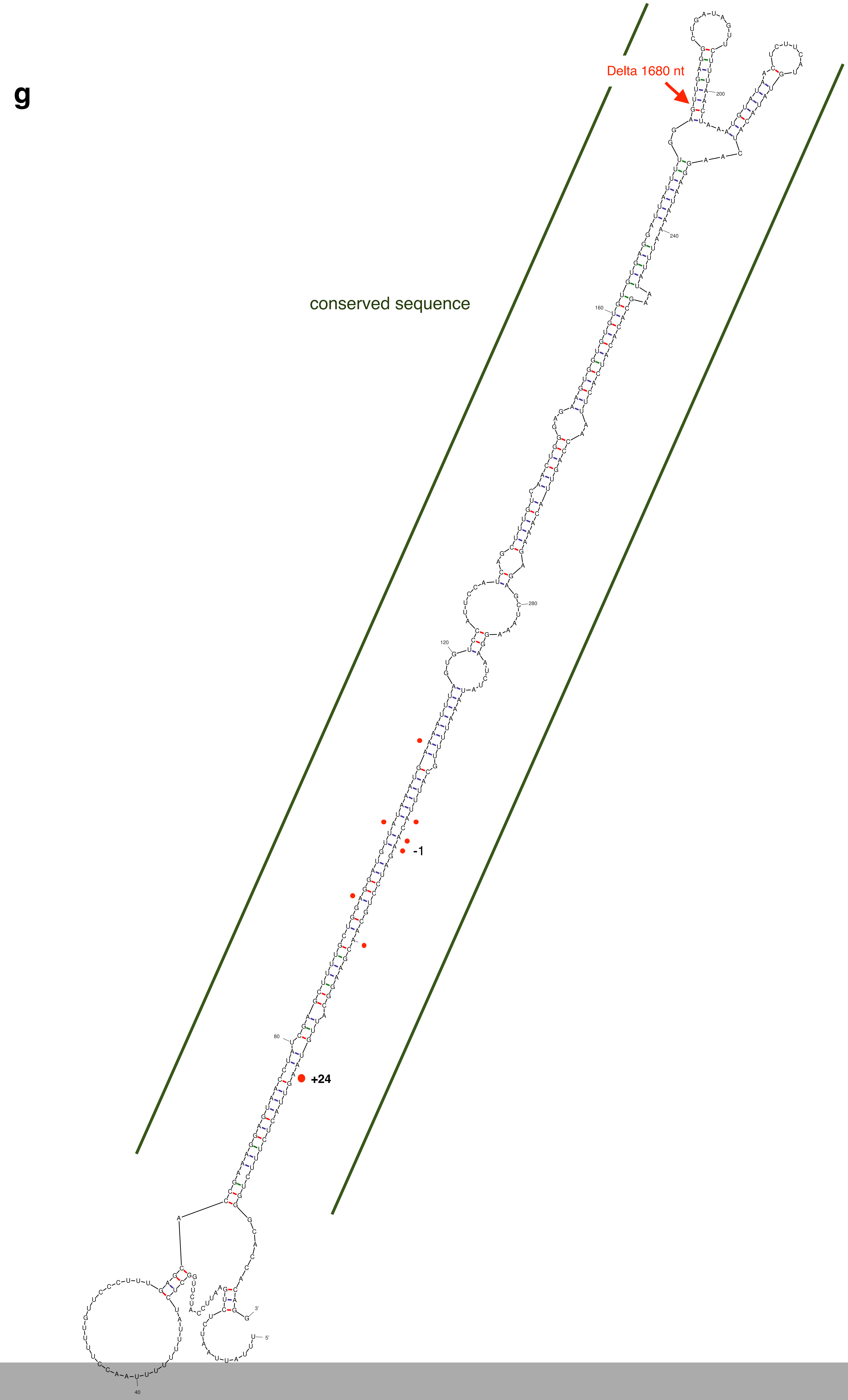

dG = -99.96 [Initially -106.70] ADARB1

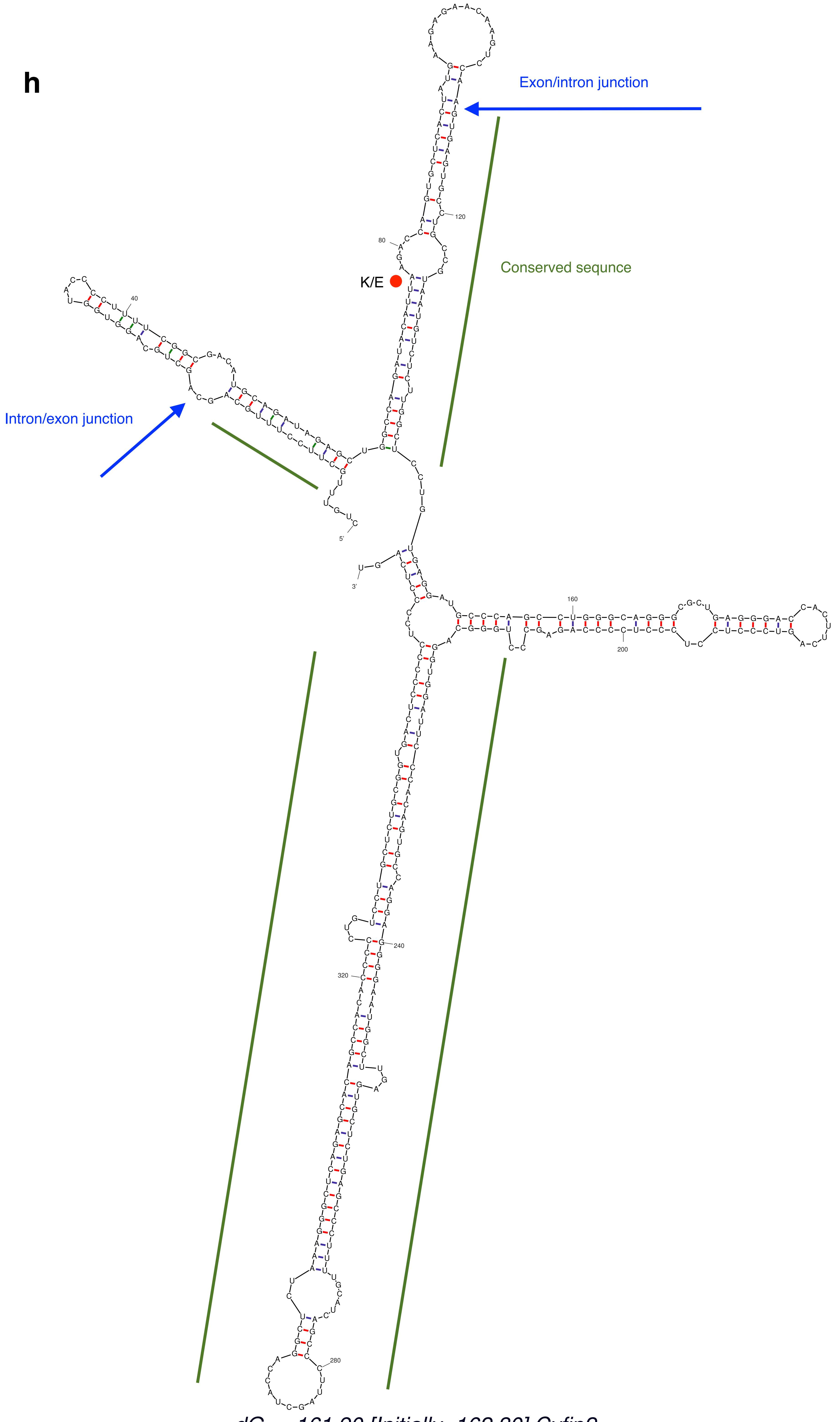

dG = -161.90 [Initially -162.30] Cyfip2

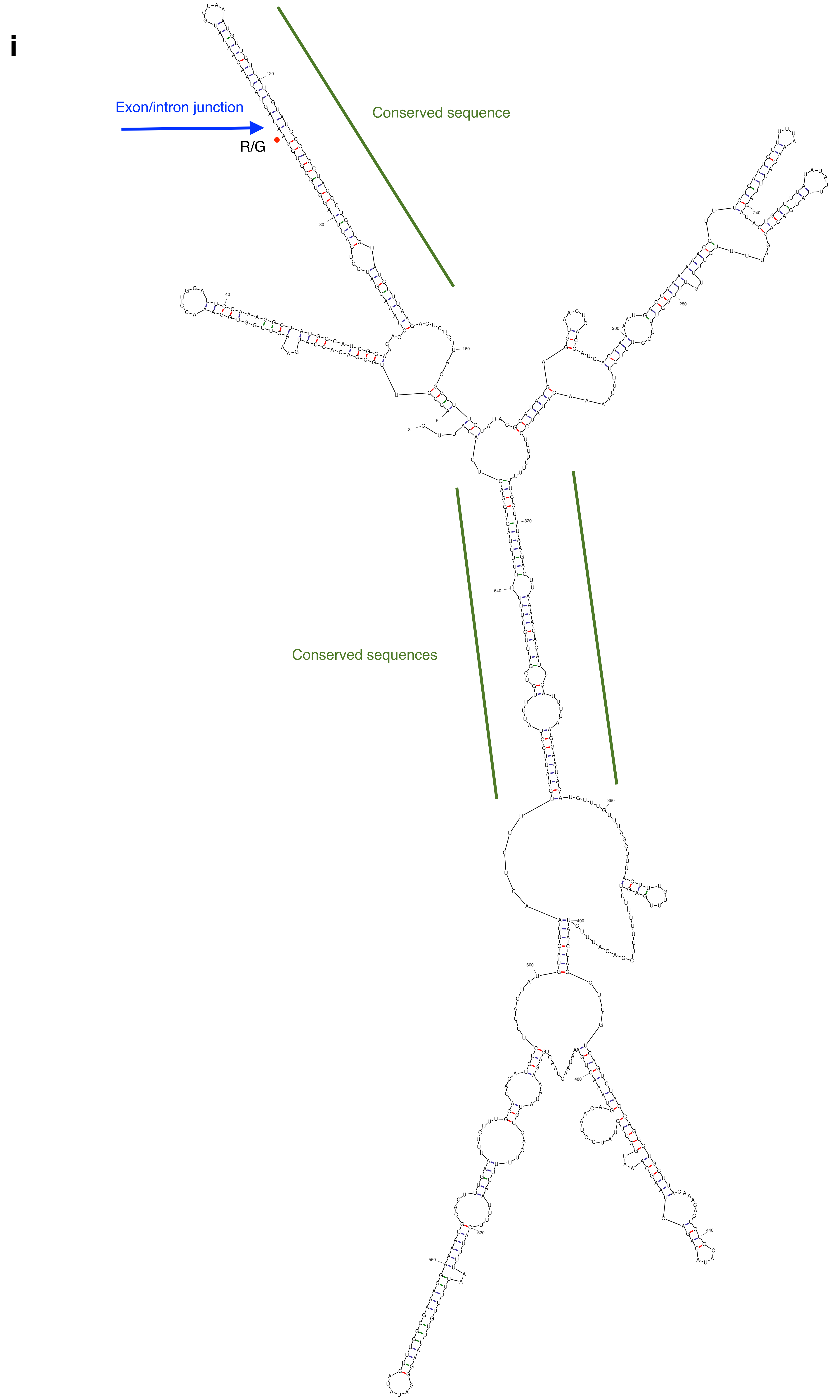

dG = -141.53 [Initially -162.80] Gria2 R/G

j

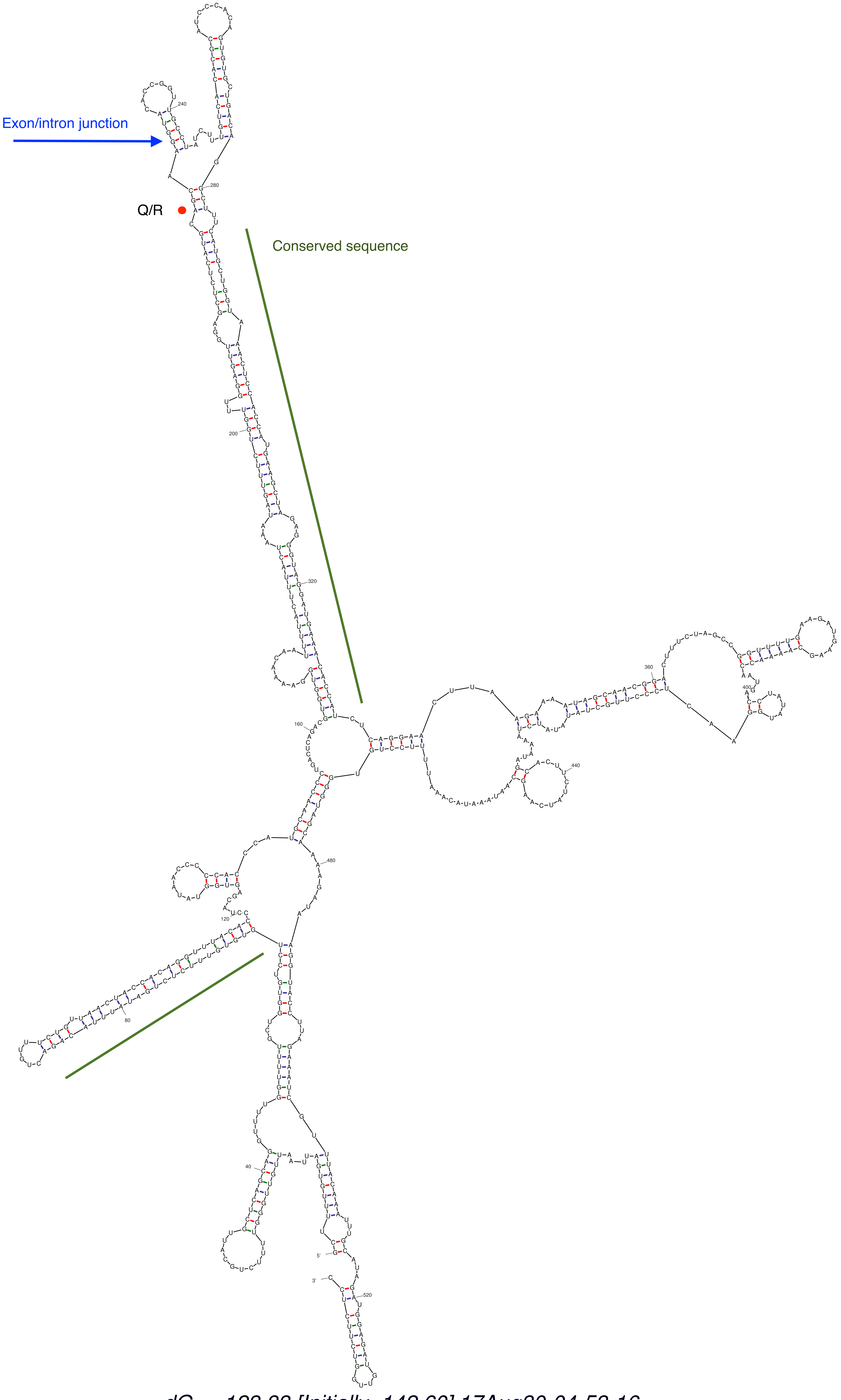

dG = -123.83 [Initially -142.60] 17Aug30-04-53-16

k

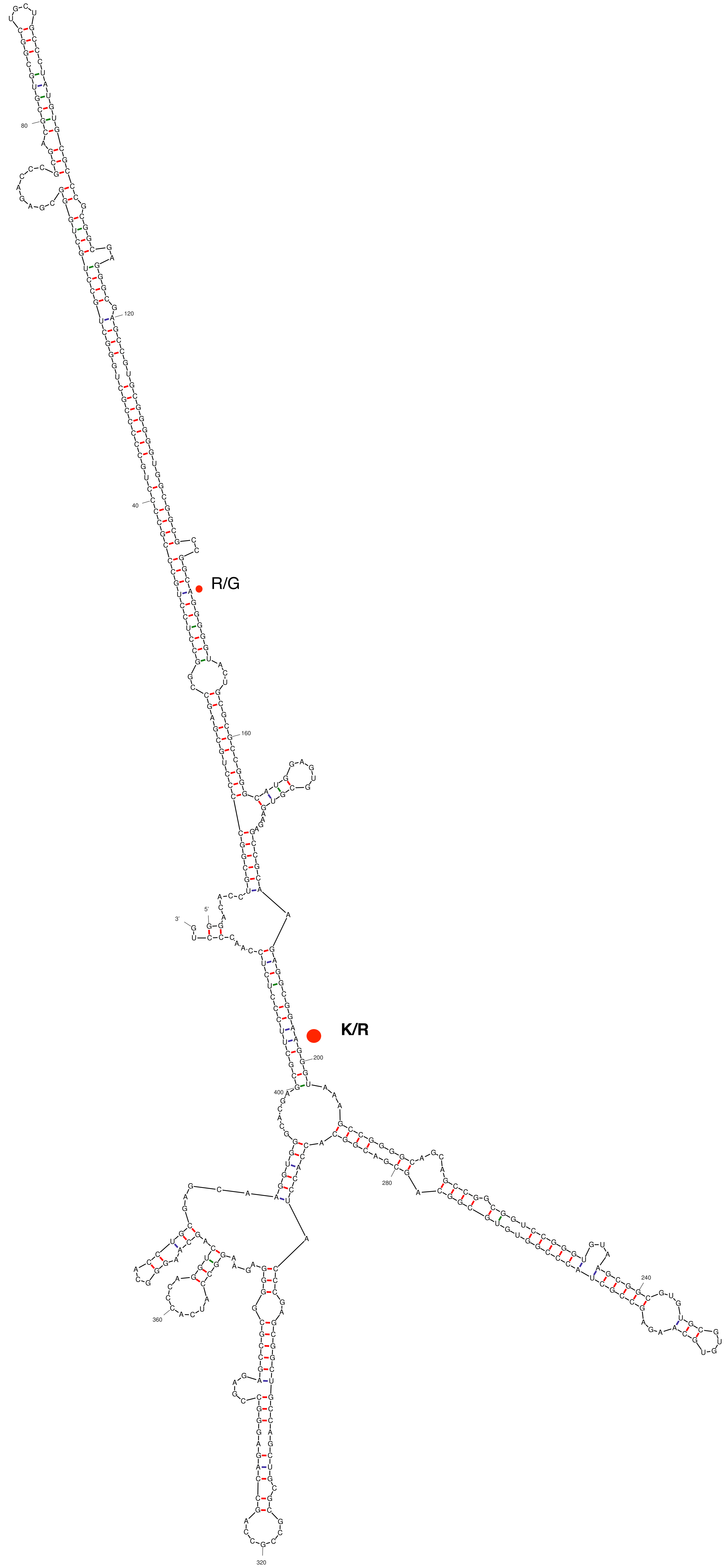

dG = -210.71 [Initially -218.40] IGFBP7

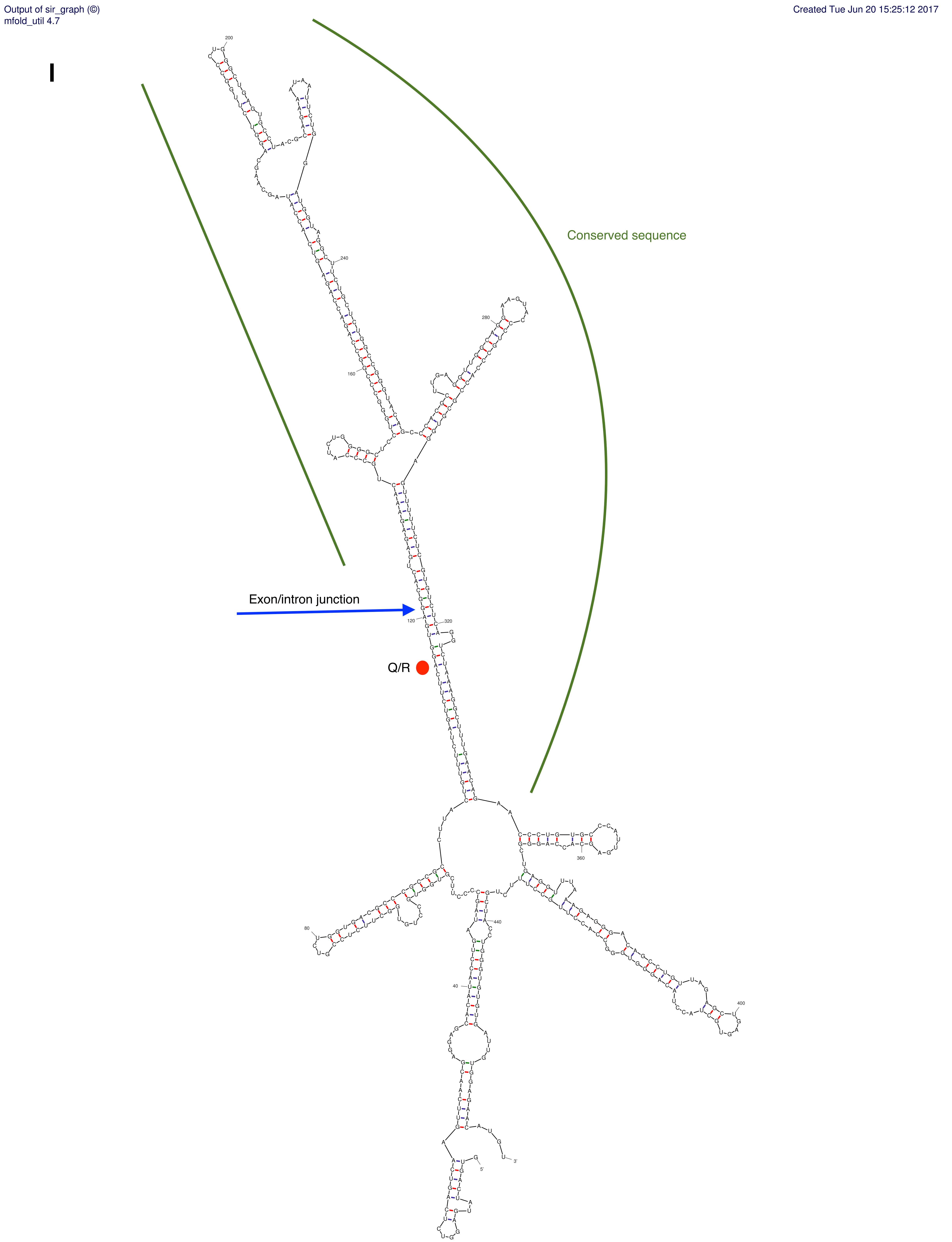

dG = -202.88 [Initially -206.80] FLNA

m

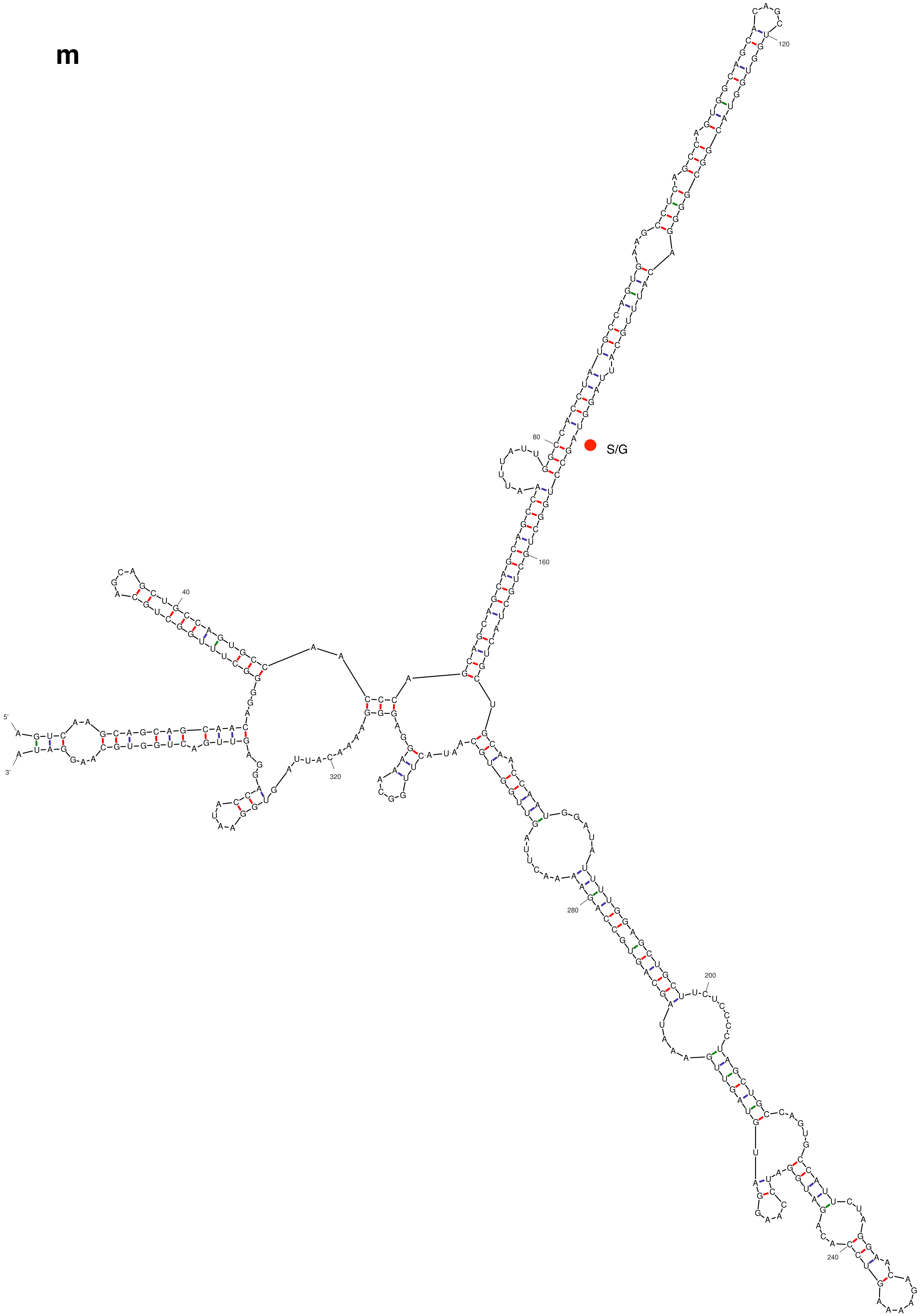

dG = -128.40 [Initially -136.00] nova1

n

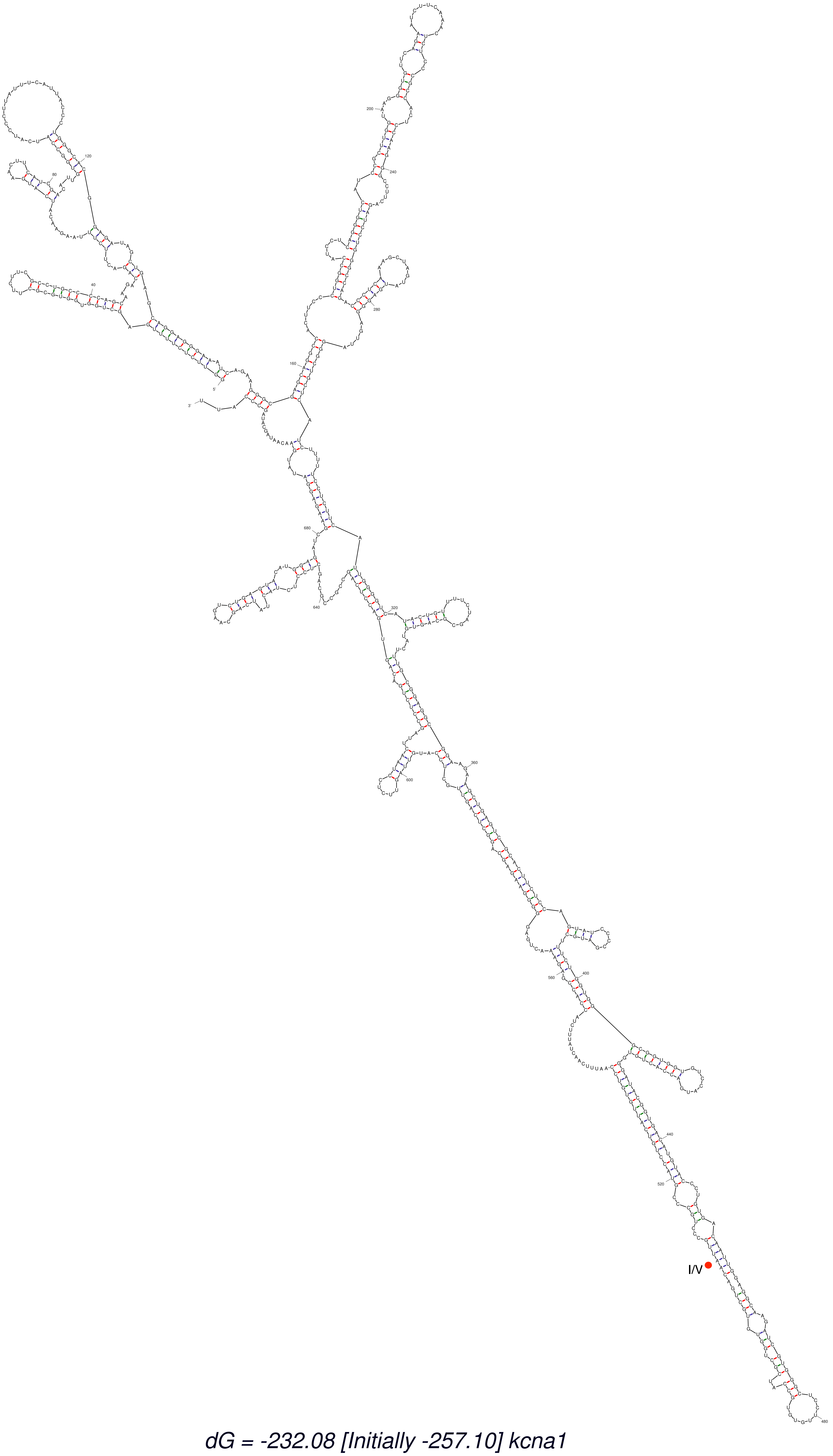

dG = -232.08 [Initially -257.10] kcna1

o

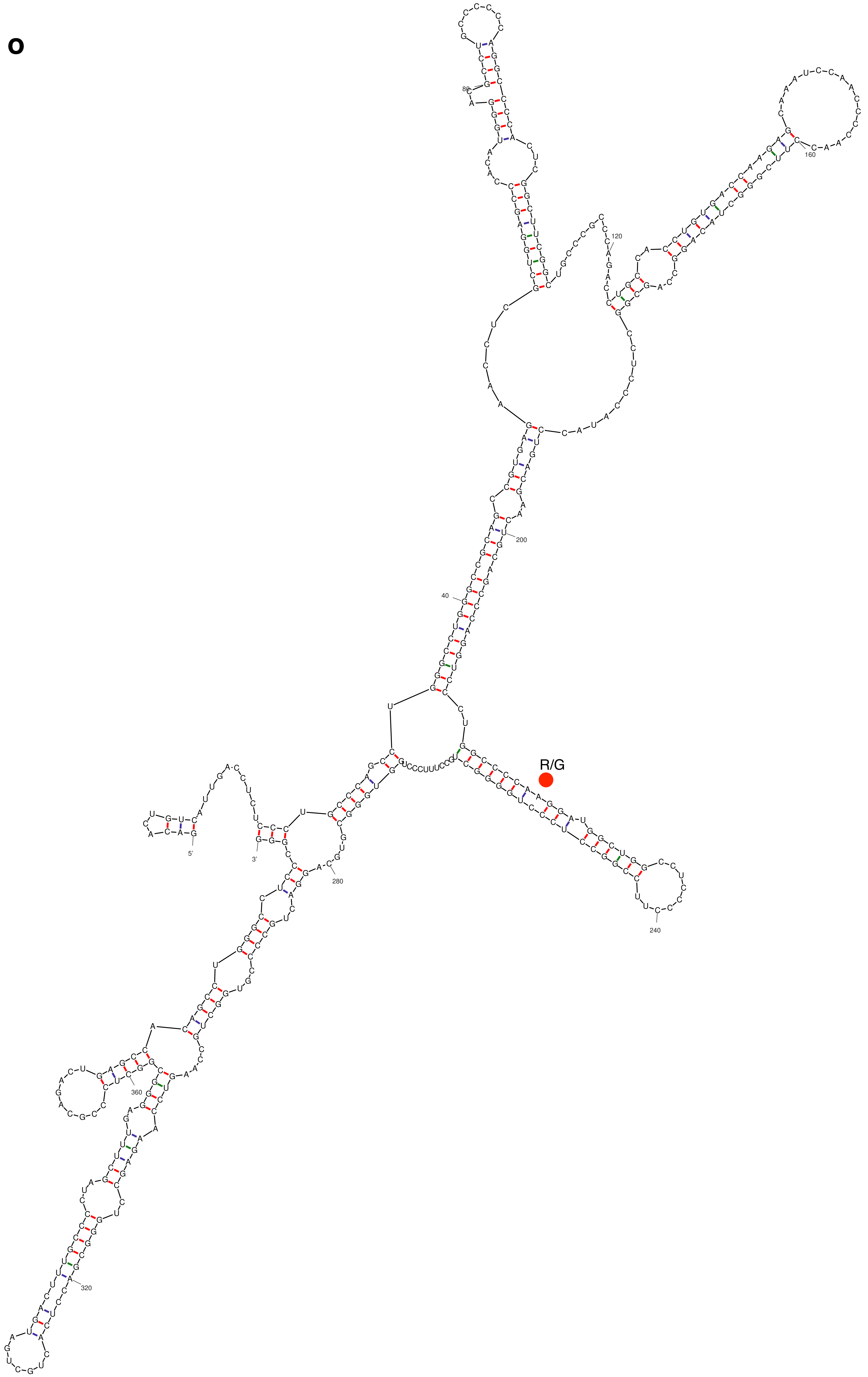

dG = -151.85 [Initially -165.10] PLCH2

p

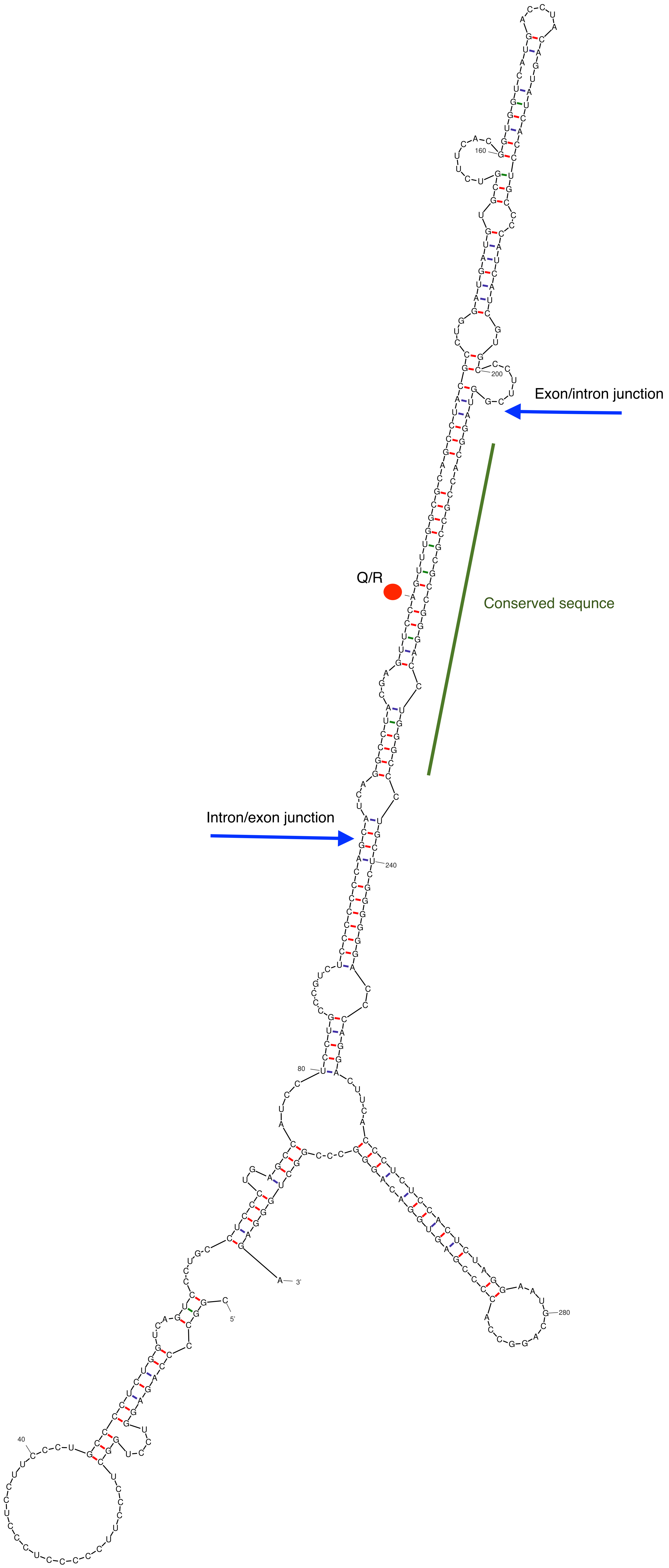

dG = -124.04 [Initially -127.60] TMEM63B

q

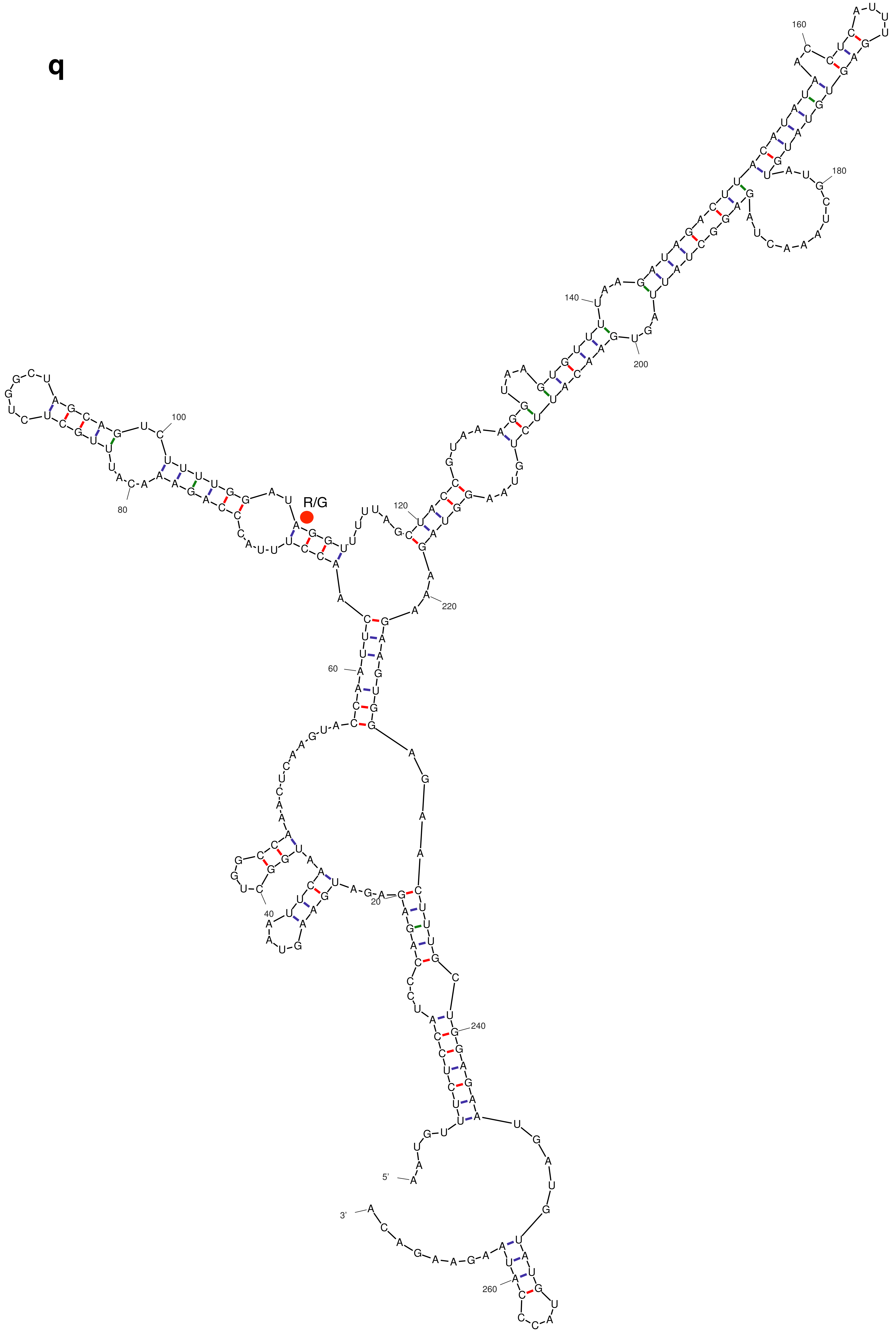

dG = -52.63 [Initially -59.40] CCNI

r

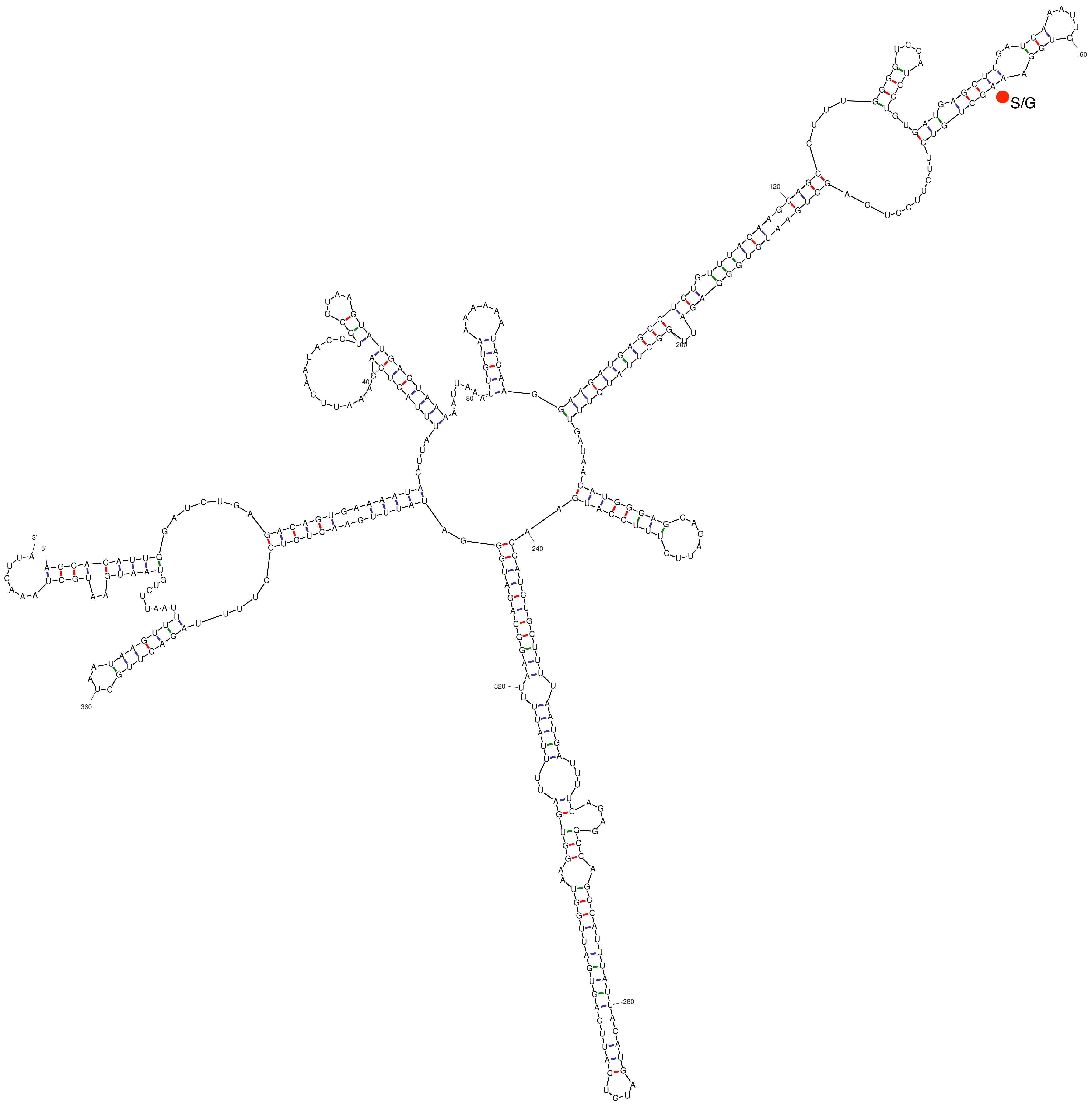

dG = -94.92 [Initially -104.30] Azin1

S

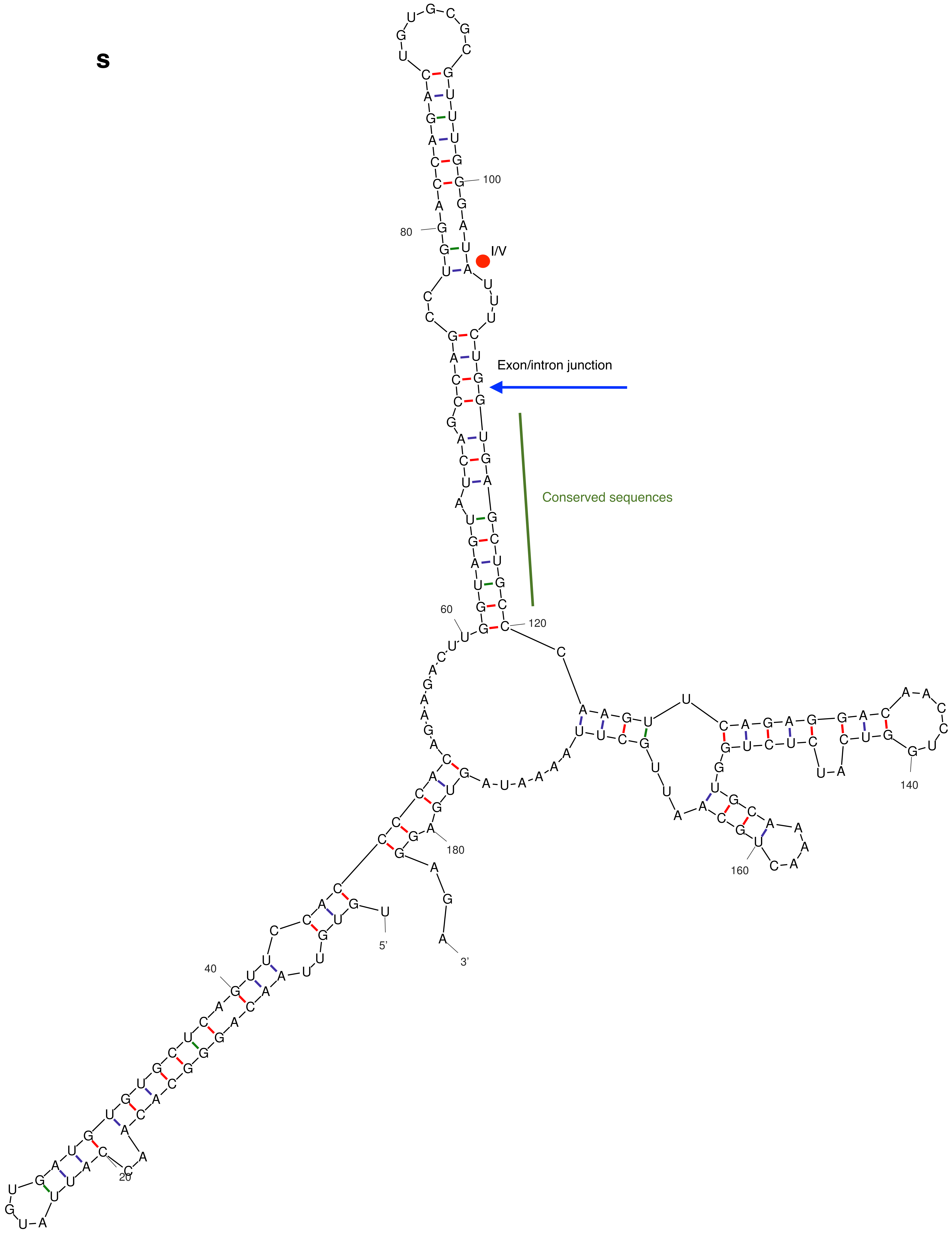

dG = -50.72 [Initially -53.30] copa

t

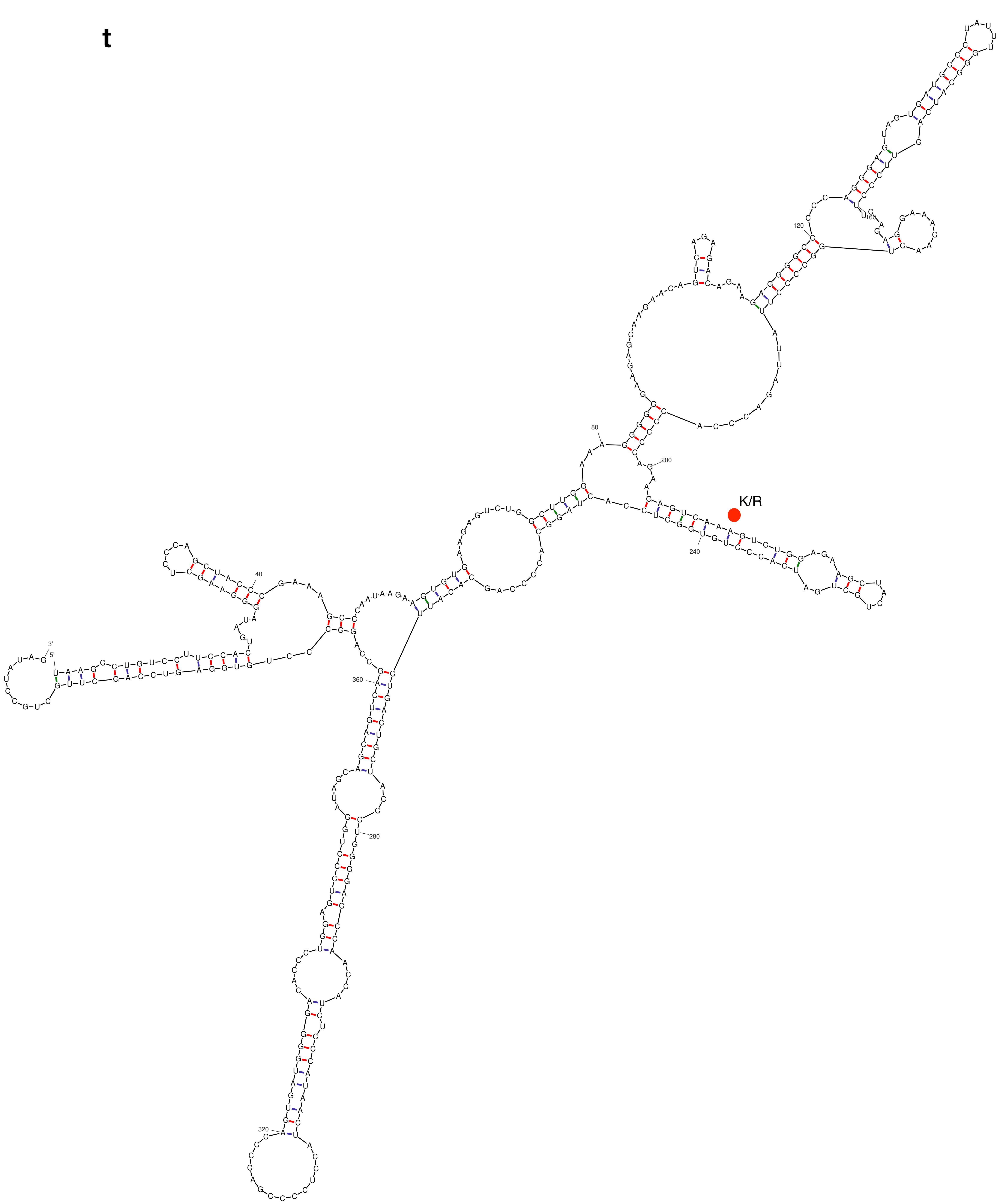

dG = -125.96 [Initially -142.20] GPATCH8

u

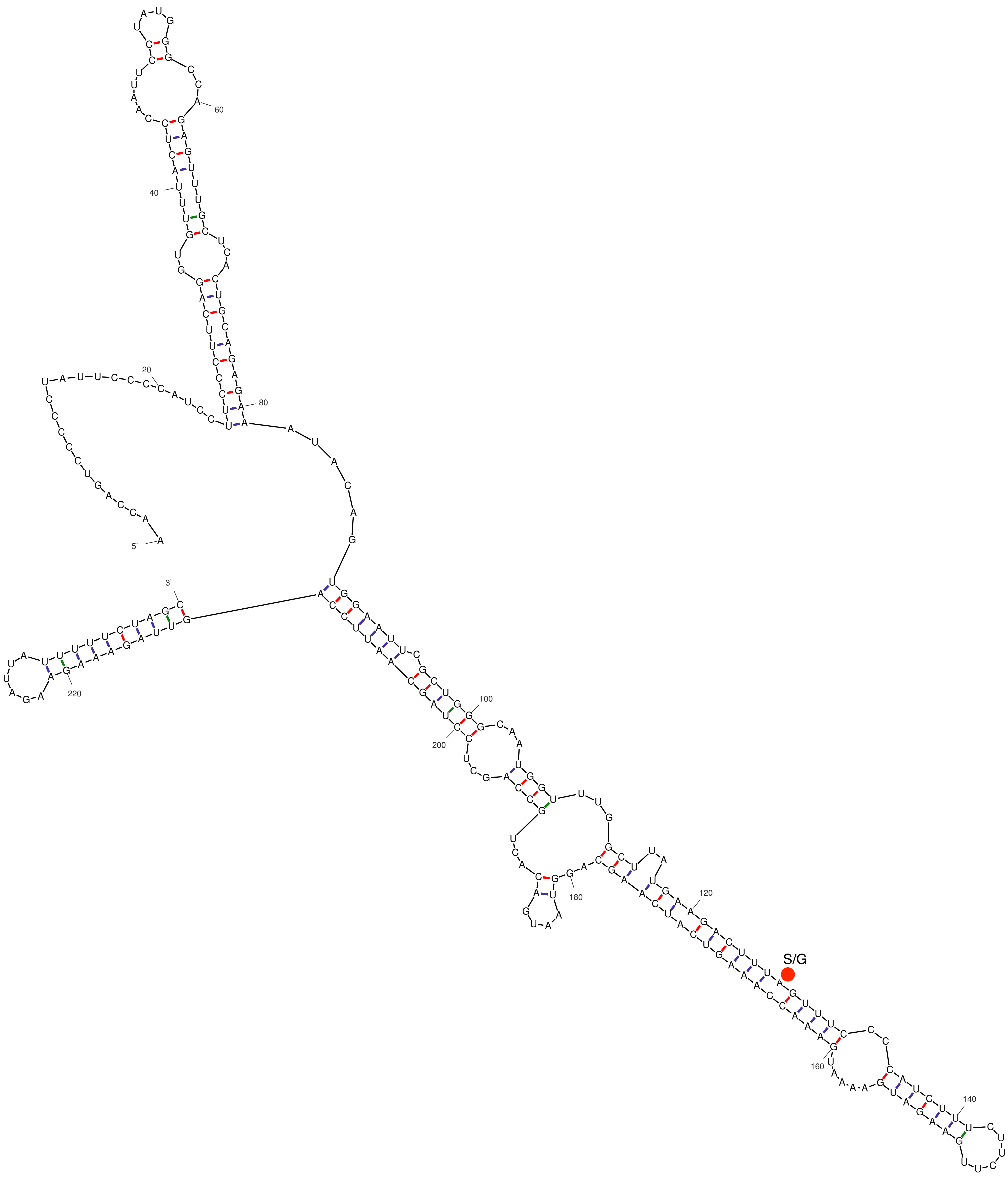

dG = -61.78 [Initially -62.30] NCSTN

v

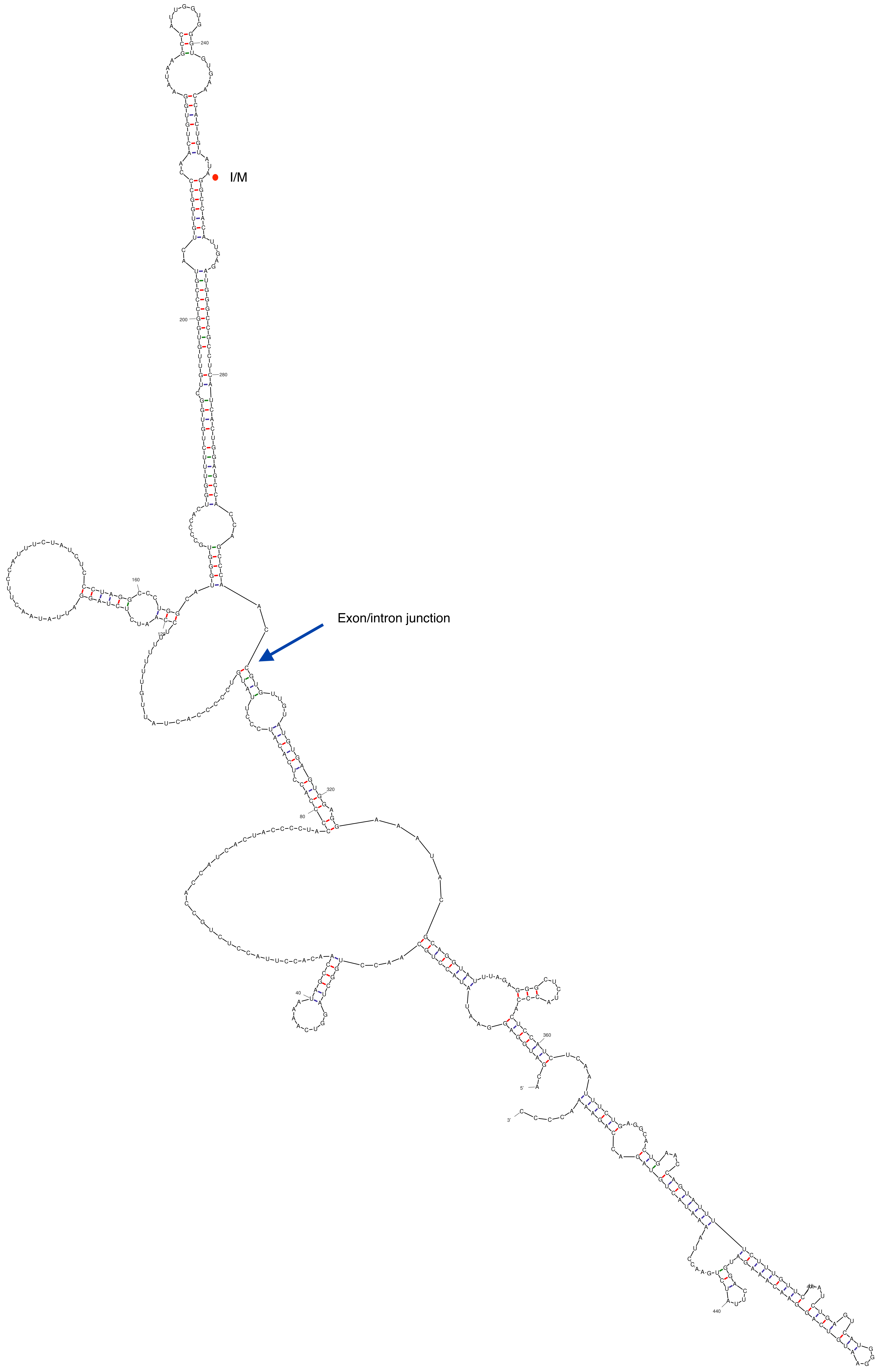

dG = -130.76 [Initially -144.60] OSGEP

Output of sir_graph (©)
mfold_util 4.7

Created Wed Aug 16 07:28:17 2017

X

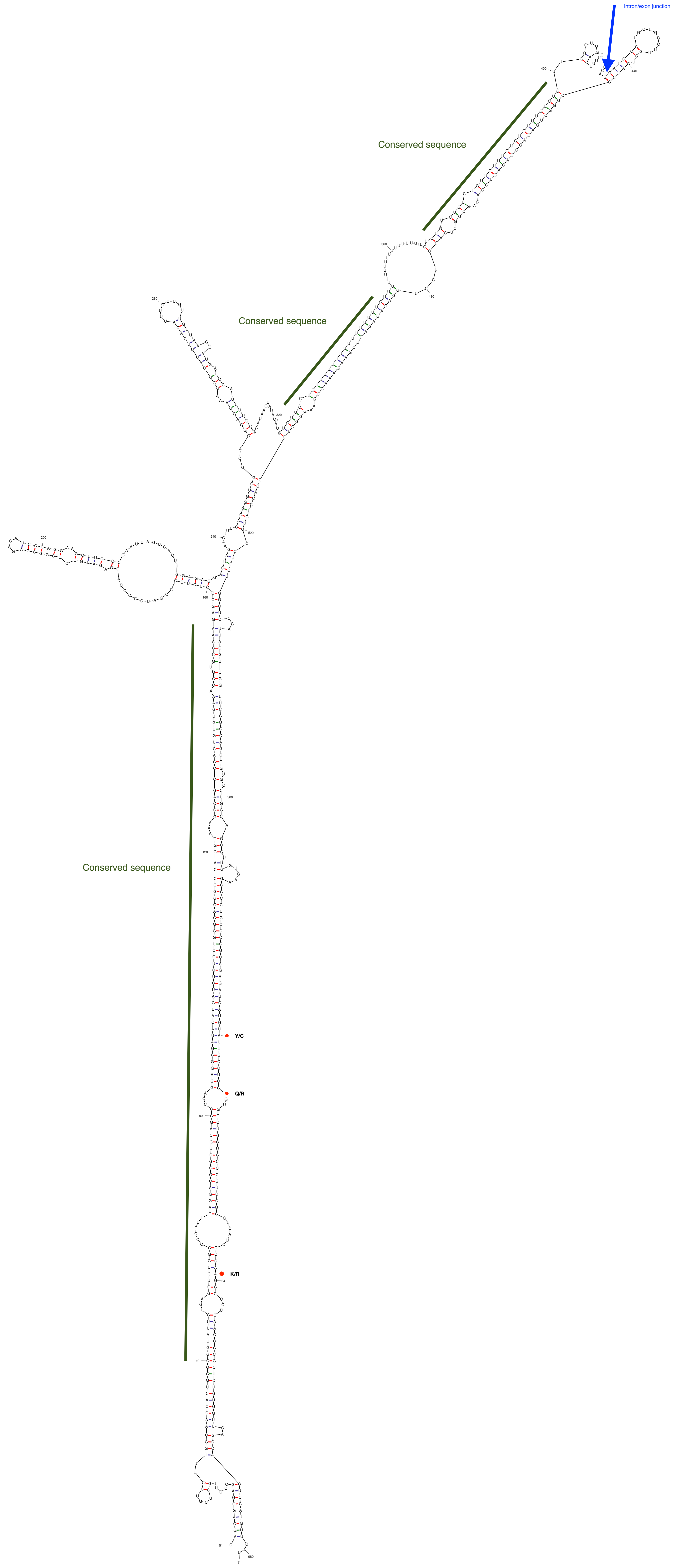

dG = -297.87 [Initially -304.50] Blcap
